# Airway immune signatures of protection and disease progression in recent human tuberculosis household contacts

**DOI:** 10.1038/s41590-026-02544-0

**Published:** 2026-06-24

**Authors:** William J. Branchett, Jee-Whang Kim, Jessica Shields, Probir Chakravarty, Jo Lee, Ismail Novsarka, Hubert Slawinski, Katalin A. Wilkinson, Robert J. Wilkinson, Anver Kamil, Raman Verma, Pranabashis Haldar, Anne O’Garra

**Affiliations:** 1 Immunoregulation and Infection Laboratory, The Francis Crick Institute, London, UK; 2 Department of Respiratory Sciences, University of Leicester, Leicester, UK; 3 Respiratory Department, University Hospitals of Leicester, Glenfield Hospital, Leicester, UK; 4 NIHR Leicester Biomedical Research Centre, Glenfield Hospital, Leicester, UK; 5 Bioinformatics and Biostatistics Science Technology Platform, The Francis Crick Institute, London, UK; 6 Genomics Science Technology Platform, The Francis Crick Institute, London, UK; 7 Tuberculosis Laboratory, The Francis Crick Institute, London, UK; 8 Department of Infectious Disease, Imperial College London, London, UK; 9 Wellcome Discovery Research Platforms in Infection, Centre for Infectious Diseases Research in Africa, Institute of Infectious Disease and Molecular Medicine and Department of Medicine, University of Cape Town, Cape Town, Republic of South Africa; 10 Nuclear Medicine Department, University Hospitals of Leicester, Glenfield Hospital, Leicester, UK

**Keywords:** Infection, Tuberculosis

## Abstract

The local immune factors dictating whether individuals who have been infected with *Mycobacterium tuberculosis* remain healthy or progress to active tuberculosis (TB) have not been defined. Here we interrogated the airway immune response at single-cell resolution in bronchoalveolar lavage from positron emission and computed tomography-characterized recent TB household contacts, who either controlled the infection or progressed to TB disease, as well as of patients with active TB at diagnosis. Single-cell RNA sequencing revealed type I IFN-dependent and IFN-independent neutrophil signatures in bronchoalveolar lavage from patients with active TB and TB progressors. We report an inverse relationship between airway neutrophils and T cells, with T cells showing signatures of exhaustion, cytotoxicity and cell death in progressors and patients with active TB with a neutrophil-dominated airway profile. Conversely, we identified T cell signatures of protection in nonprogressor contacts dominated by genes related to regulation, quiescence and a stem-like profile. Our findings from early human airway responses in TB contacts reveal genes, pathways and cell states that may dictate infection outcome and inform strategies for developing effective host-directed therapies and vaccines.

## Abstract (web summary)

The local immune factors dictating whether individuals who have been infected with *Mycobacterium tuberculosis* remain healthy or progress to active tuberculosis (TB) have not been defined. Here O’Garra and colleagues analyze early human airway responses in TB household contacts to reveal immune signatures of protection and disease progression.

## Main

A quarter of the global population is estimated to have been infected with *Mycobacterium tuberculosis*, the causative pathogen of tuberculosis (TB). However, only 5–10% of infected individuals progress to active TB^1^, typically within 1–3 years^2,3^. The early local immune factors that determine protection or disease progression are unclear. Bacillus Calmette–Guérin (BCG), the only licensed TB vaccine, offers limited protection against pulmonary TB, highlighting the need to better understand mechanisms of immune protection against human TB^4–6^. Clinically, the antigen-specific interferon (IFN)-γ release assay (IGRA), is used to measure peripheral memory T cell responses to *M. tuberculosis*; however, this does not distinguish current from past infection or predict progression to active TB. Detection of subclinical lung pathology by ^18^F-fluorodeoxyglucose positron emission and computed tomography (PET–CT), which reveals highly metabolically active cells, has better positive predictive value for clinical progression, consistent with evolving pulmonary and intrathoracic nodal inflammatory changes during progression^7–10^. Blood transcriptomics have identified neutrophil-driven type I IFN-inducible signatures in active TB that correlate with disease severity and progression, supporting a role for this pathway in failed immunity and immunopathology of TB^3,11,12^. The role of type I IFN in exacerbated TB disease is supported by studies in experimental models^13–17^. Control of *M. tuberculosis* is dependent on T-cell-mediated immunity^18^, but the precise mechanisms underpinning this are unclear^5,6^.

Key questions remain regarding: (1) the mechanisms of failed immune control of *M. tuberculosis*, which could inform strategies for host-directed immunomodulating therapies and (2) the early, local, immune signatures associated with control of *M. tuberculosis* infection, which could provide correlates of protection and strategies for improving TB vaccines. Here, we performed in-depth studies of the lower-airway immune response in prospective cohorts of treatment-naive patients with active TB and recent household TB contacts to reveal the primary immune events in close proximity to the site of *M. tuberculosis* infection, accompanying protection or disease progression. The study was conducted in Leicester (UK), a low TB-burden setting, lowering the risk of confounding *M. tuberculosis* reinfection that affects high TB-burden settings, during prospective evaluation of outcome^19^. Single-cell RNA sequencing (scRNA-seq), bulk RNA-seq and flow cytometry analysis of bronchoalveolar lavage (BAL) were integrated with clinical parameters and PET–CT to reveal immune signatures at single-cell resolution and how these related to a spectrum of TB progressor and nonprogressor states.

## Results

### Characterizing the airway immune response in patients with TB and contacts

**Fig. 1:**
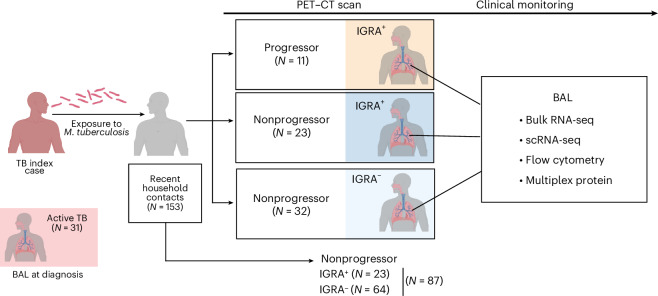
Schematic of the study on patients with TB and their household contacts. A total of 153 recent household contacts of active pulmonary TB patients were recruited, of whom 11 progressed to TB disease (progressors). Bronchoalveolar lavage (BAL) was sampled from the 11 progressors and 55 contacts who did not progress (nonprogressors). Further details are available in the Methods, Extended Data Fig. 1a,b and Supplementary Tables 1,a–c. Illustration created in BioRender; Branchett, W. https://biorender.com/uhc5llr (2026).

**Extended Data Fig. 1:**
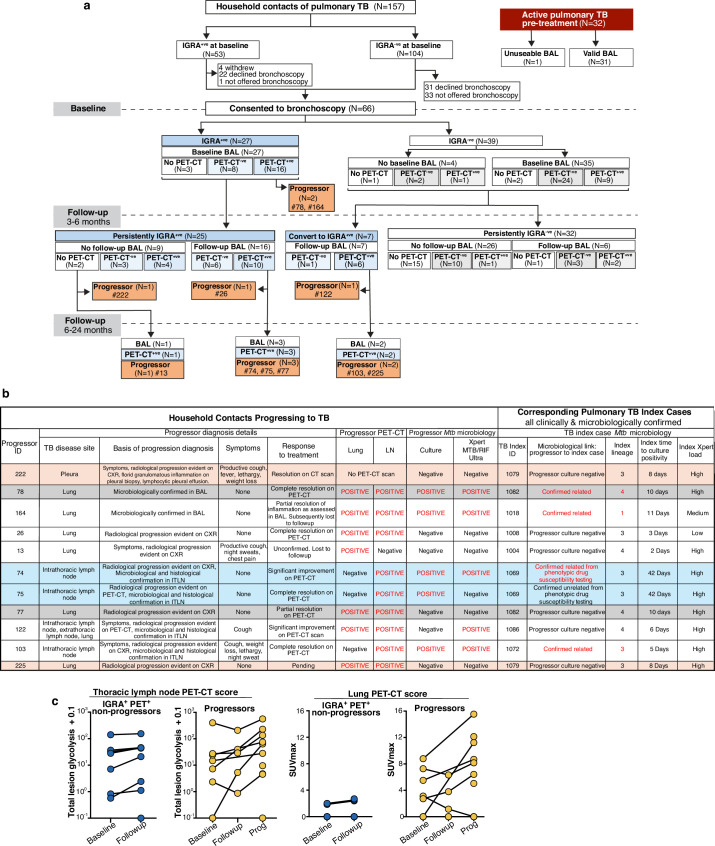
Study overview and details on progressors. **a**, Expanded flow chart showing recruitment, sampling points and TB progression events in the study. **b**, Details on contacts who progressed to TB and their respective TB index cases. Colored rows indicate progressor contacts of the same index case. ITLN, intrathoracic lymph node. **c**, PET^−^CT scores from thoracic lymph node and lung in progressors and IGRA^+^ non-progressors presenting with positive thoracic PET-CT. Total lesion glycolysis (TLG) represents combined metabolic activity from all positive lymph nodes (SUV_mean_ x metabolic volume). SUV_max_ was determined from the highest avid lung lesion. Data are shown from baseline and follow-up visits and, where applicable, at progression, with lines connecting individuals. See also Supplementary Table 1 and PET-CT scan images in Supplementary Fig. 1.

To identify the early airway events that may dictate whether individuals exposed to patients with active pulmonary TB progressed to TB or remained healthy, 153 household contacts were recruited, of whom 11 progressed to TB disease (progressors), consistent with expected rates^20^ (Fig. 1, Extended Data Fig. 1a,b and Supplementary Table 1a,c). Of those consenting to bronchoscopy, we obtained BAL samples from the 11 progressors and 55 contacts who did not progress, termed nonprogressors (23 IGRA-positive (IGRA^+^) and 32 persistently IGRA-negative (IGRA^−^)) (Fig. 1, Extended Data Fig. 1a,b and Supplementary Table 1,a,c). Progression was accompanied by a positive and increasing thoracic PET–CT signal, whereas a stable positive PET–CT signal was detected in a subset of IGRA^+^ but not IGRA^−^ nonprogressor contacts (Extended Data Fig. 1c, Supplementary Table 1a and Supplementary Fig. 1a).

**Extended Data Fig. 2:**
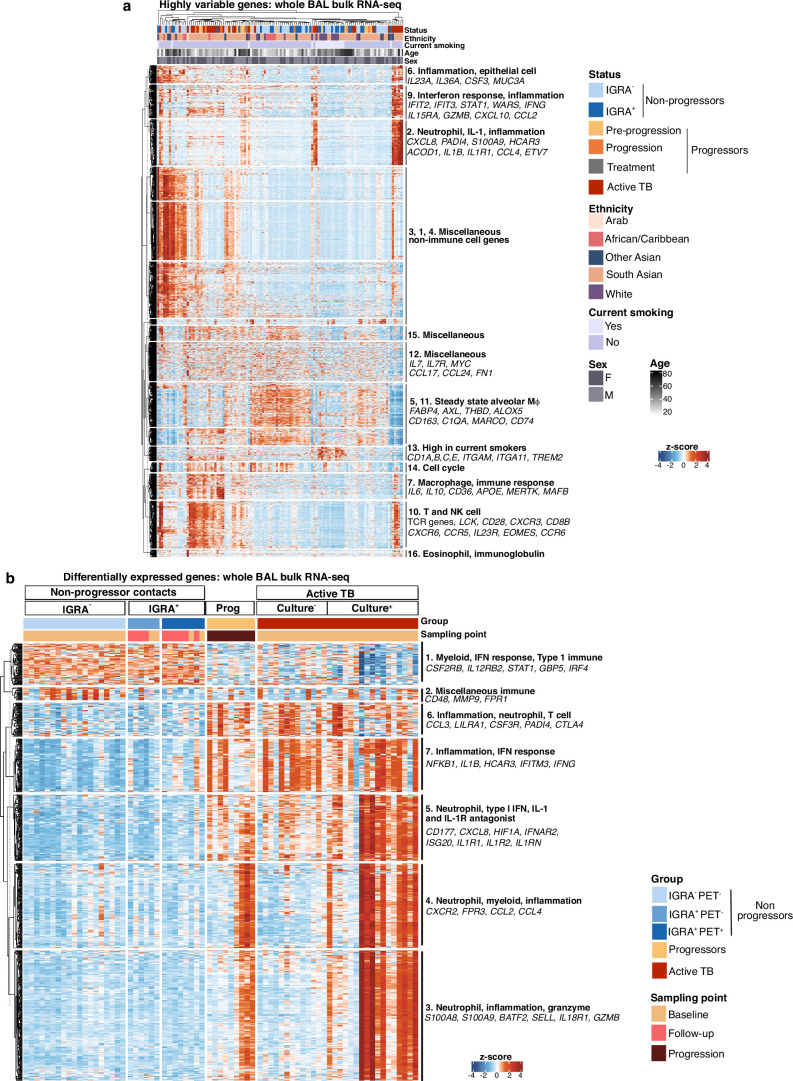
Global transcriptional changes in airway cells of TB patients and household contacts by bulk RNA^−^seq. **a**, Unsupervised Ward’s clustering of highly variable genes among all BAL samples for which bulk RNA-seq was obtained, before excluding any outliers and including multiple timepoints for individuals, where available (Supplementary Table 1,a-b). **b**, k-means clustering of total differentially expressed genes combined from comparisons between i) IGRA^+^ PET-CT^−^(N = 6) and IGRA^−^PET-CT^−^(N = 19) non-progressors; ii) IGRA^+^ PET-CT^+^ (N = 8) and IGRA^−^PET-CT^−^non-progressors and iii) IGRA^+^ PET-CT^+^ non-progressors and progressors at progression (N = 9); clustered across samples from all groups, including BAL culture-positive (N = 13) and culture-negative (N = 17) active TB, after excluding outlier samples as detailed in (Methods and Supplementary Tables 1a,b). In both panels, cluster numbers are stated and representative genes and associated pathways and cell types from each cluster are listed (see also Supplementary Tables 2 and 3).

BAL was subjected to bulk and scRNA-seq, flow cytometry and multiplex protein assays. All BAL data were compared with those from patients with active pulmonary TB at diagnosis (Fig. 1, Extended Data Fig. 1a and Supplementary Table 1b). Unbiased analysis of BAL bulk RNA-seq distinguished patients with TB and progressing contacts from nonprogressors and was not skewed by age, sex or ethnicity (Extended Data Fig. 2a and Supplementary Table 2). Expression of genes related to inflammation, T cell and neutrophil functions was increased in BAL from progressors and patients with active TB (Extended Data Fig. 2b and Supplementary Table 3).

### Opposing neutrophil and T cell responses in both patients with TB and progressors

**Fig. 2:**
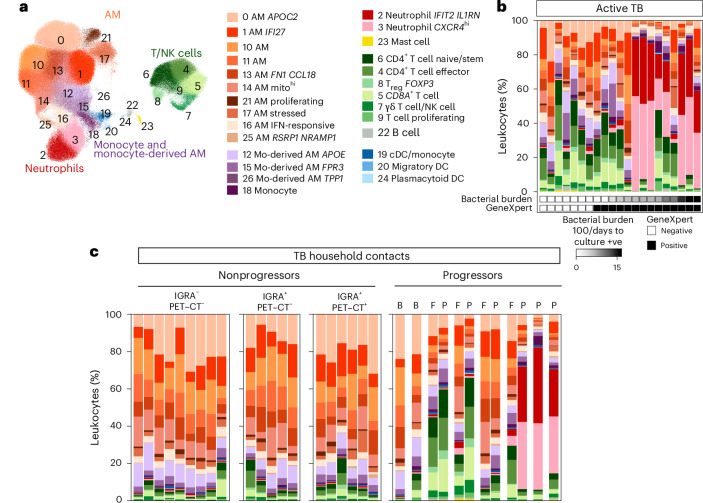
scRNA-seq reveals the local airway immune response in patients with TB and household contacts. **a**, UMAP visualization of total leukocytes (894,159 cells) from BAL samples of all patients with TB and household contacts (total 77 samples from 62 individuals). **b**,**c**, Stacked bar plots showing percentages of cells in each scRNA-seq cluster per participant. Mo-derived, monocyte-derived; cDC, conventional dendritic cell. **b**, Patients with active TB (*N* = 21) are ordered by increasing BAL *M. tuberculosis* bacterial burden (100 divided by days to culture positivity (+ve), with a value of 0 indicating negative BAL culture), with *M. tuberculosis* GeneXpert (Xpert MTB/RIF Ultra) results also reported. **c**, Nonprogressor contacts are grouped on the basis of IGRA and PET–CT status after excluding clinical and radiographic outliers: IGRA^−^ PET–CT^−^(*N* = 9), IGRA^+^PET–CT^−^ (*N* = 5), IGRA^+^PET–CT^+^ (*N* = 6), progressors (*N* = 8). Each column represents an individual contact, with the latest available time point after baseline shown. Where available for progressors, preprogression and progression samples are shown. B, baseline; F, follow-up; P, progression.

**Fig. 3:**
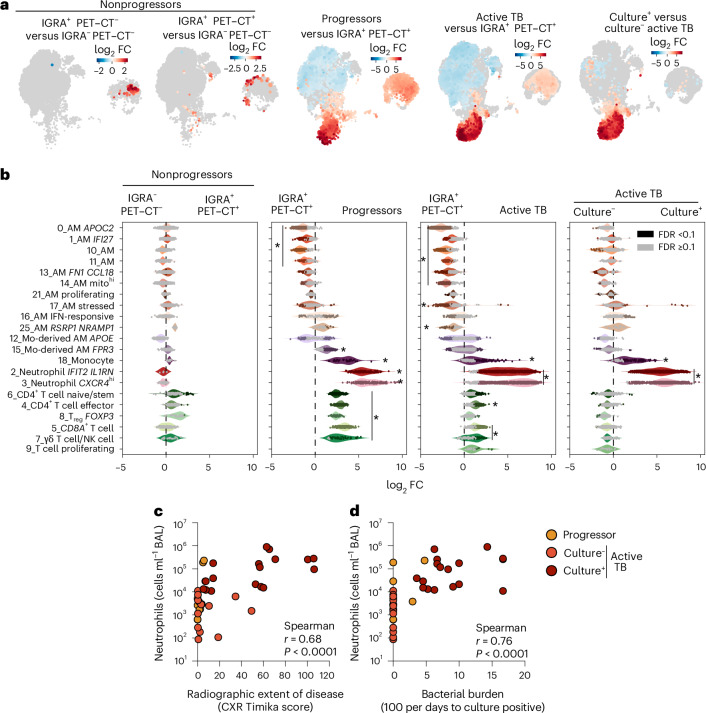
Airway neutrophil increase with TB disease progression. **a**,**b**, Milo differential abundance analysis of scRNA-seq cell clusters compared between the indicated groups, as detailed in the Methods. IGRA^−^ PET–CT^−^(*N* = 9), IGRA^+^PET^−^CT^−^ (*N* = 5) and IGRA^+^PET–CT^+^ (*N* = 6) nonprogressors and progressors at progression (*N* = 6); TB patients with negative BAL *M. tuberculosis* culture (*N* = 9) and TB patients with positive BAL *M. tuberculosis* culture (*N* = 12). Differentially abundant cell neighborhoods are colored by log_2_ FC between groups and superimposed onto UMAPs (**a**). Cell neighborhoods were superimposed onto corresponding BAL leukocyte clusters as defined in Fig. 2a (**b**). Each dot represents one neighborhood, with FC and false discovery rate (FDR) results for differential abundance indicated. Asterisks mark clusters for which ≥20% of assigned neighborhoods were significantly differentially abundant in a consistent direction (FDR <0.1) between the indicated groups. **c**,**d**, Neutrophil counts in progressors (*N* = 10) and patients with BAL culture-negative (*N* = 13) and -positive (*N* = 17) TB, as determined by flow cytometry, plotted per patient against chest X-ray Timika score (**c**) or estimated BAL *M. tuberculosis* bacterial burden (100 divided by days to culture positivity, with a value of 0 indicating negative BAL *M. tuberculosis* culture) (**d**). Results of Spearman correlation analysis (two tailed) are shown. Culture^+^, culture-positive; Culture^−^, culture-negative.

**Extended Data Fig. 3:**
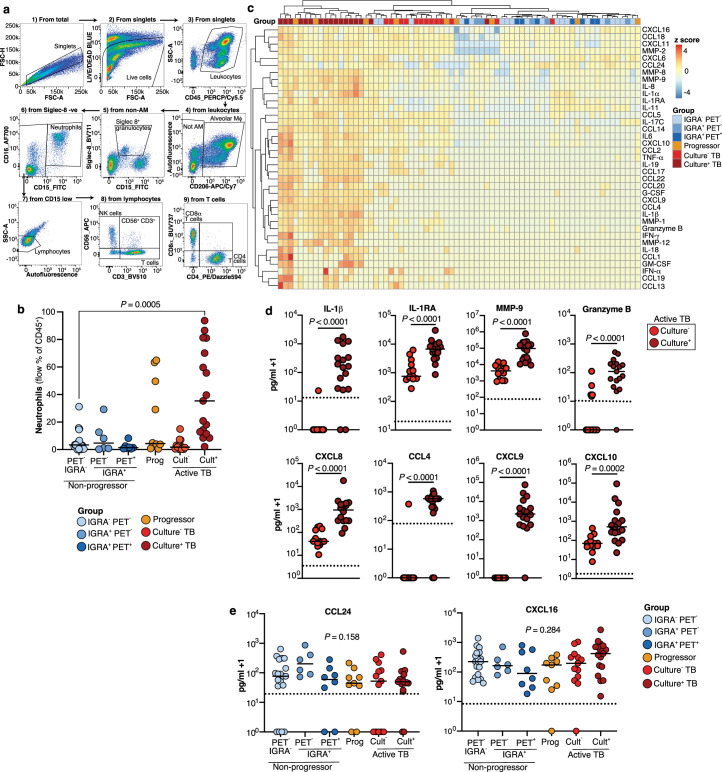
Bronchoalveolar lavage flow cytometry and protein multiplex assays. **a**, Flow cytometry gating strategy for the pan-immune cell panel used to quantify neutrophils (CD15^hi^ CD16^+^ CD206^low−neg^). **b**, Percentage of neutrophils within total live CD45^+^ BAL leukocytes as determined by flow cytometry. Points show data from individual participants with lines at the median; IGRA^−^ PET-CT^−^(N = 19), IGRA^+^ PET-CT^−^ (N = 6), IGRA^+^ PET-CT^+^ (N = 7), Progressors at progression (N = 10); BAL culture^−^ TB (N = 13) BAL culture^+^ TB (N = 17). Statistics: Kruskal-Wallis with Dunn’s *post hoc* test (two-tailed). (**c)** Row-scaled heat-map showing Ward’s clustering of samples based on analyte concentrations in BAL supernatants as determined by Luminex multiplex immunoassays. **d-e**, Concentrations of selected protein analytes in BAL supernatants, with dashed lines indicating the effective limit of detection. Values of zero indicate no detection above this limit. Points show data from individual participants with lines at the median. IGRA^−^PET-CT^−^(N = 19), IGRA^+^ PET-CT^−^ (N = 6), IGRA^+^ PET-CT^+^ (N = 8), Progressors at progression (N = 9) and BAL culture-negative (N = 13) and culture-positive (N = 17) TB patients. Statistics: (**d**) Mann-Whitney test (two-tailed); ***, *P* < 0.001; ****, *P* < 0.0001 (**e**) Kruskal-Wallis test.

To further probe the molecular and cellular changes in airway cells of patients with TB and their contacts, scRNA-seq analysis was performed on 894,159 total BAL leukocytes. Integrated analysis of the combined study groups revealed clusters consistent with alveolar macrophages (AMs), monocyte-derived macrophages, monocytes, conventional dendritic cells (DCs), two clusters of neutrophils and six clusters comprising T and natural killer (NK) cells (Fig. 2a and Supplementary Tables 4 and 5). Bulk RNA-seq, scRNA-seq and flow cytometry data revealed airway immune heterogeneity among patients with active TB, with neutrophils dominating in around 40% of patients with TB, all of whom had positive *M. tuberculosis* cultures from BAL (Fig. 2b, Extended Data Figs. 2 and 3a,b and Supplementary Tables 1b and 5), while those low in neutrophils were generally high in T cells and associated signatures (Fig. 2b, Extended Data Fig. 2 and Supplementary Table 5). BAL from half of the progressors sampled at progression was also dominated by neutrophils, with lower T cell frequency, while the remaining progressors showed elevated T cells and few neutrophils (Fig. 2c, Extended Data Fig. 3a,b and Supplementary Table 5). BAL of nonprogressing contacts mostly contained AMs (Fig. 2c and Supplementary Table 5), consistent with a largely noninflammatory airway environment in these contacts who remained healthy. Differential abundance analysis of scRNA-seq data from IGRA^+^PET–CT^−^ and IGRA^+^PET–CT^+^ (where PET–CT^−^ and PET–CT^+^ represent negative on both thoracic LN and lung PET–CT and positive on thoracic LN and/or lung PET–CT, respectively), compared with IGRA^−^ nonprogressors, showed increases only within distinct T cell clusters but no increase in neutrophils (Fig. 3a,b). Compared with IGRA^+^PET–CT^+^ nonprogressors, the progressor group exhibited increased abundance of neutrophils and increases across the breadth of T/NK cell clusters (Fig. 3a,b). In patients with active TB, there was an even greater increase in neutrophils but a less substantial increase in T/NK cell clusters, reflecting the marked dominance of neutrophils over T cells in culture-positive TB as compared with patients with culture-negative TB (Fig. 3a,b). An increase in monocytes accompanied neutrophil accumulation during TB progression (Fig. 3b). BAL neutrophil counts by flow cytometry correlated with radiographic extent of disease and bacterial burden in patients with active TB (Fig. 3c,d), consistent with neutrophil accumulation in more severe TB disease. Accordingly, BAL supernatants of patients with culture-positive TB contained high concentrations of mediators associated with inflammation and neutrophil responses, including interleukin (IL)-1β, IL-1 receptor antagonist (IL-1RA), MMP-9 and the neutrophil chemokine CXCL8, while the chemokines CCL24 and CXCL16 were found at similar concentrations across all groups (Extended Data Fig. 3c–e). It is notable that the inverse relationship observed between neutrophil and T cell frequencies in the BAL of human patients with TB and progressors is consistent with the functional opposition demonstrated between these cell types in experimental mouse models^21,22^, supporting their validity for study of immunological mechanisms of TB progression and pathology.

### Type I IFN-dependent and -independent neutrophil states increase with TB progression and severity

**Fig. 4:**
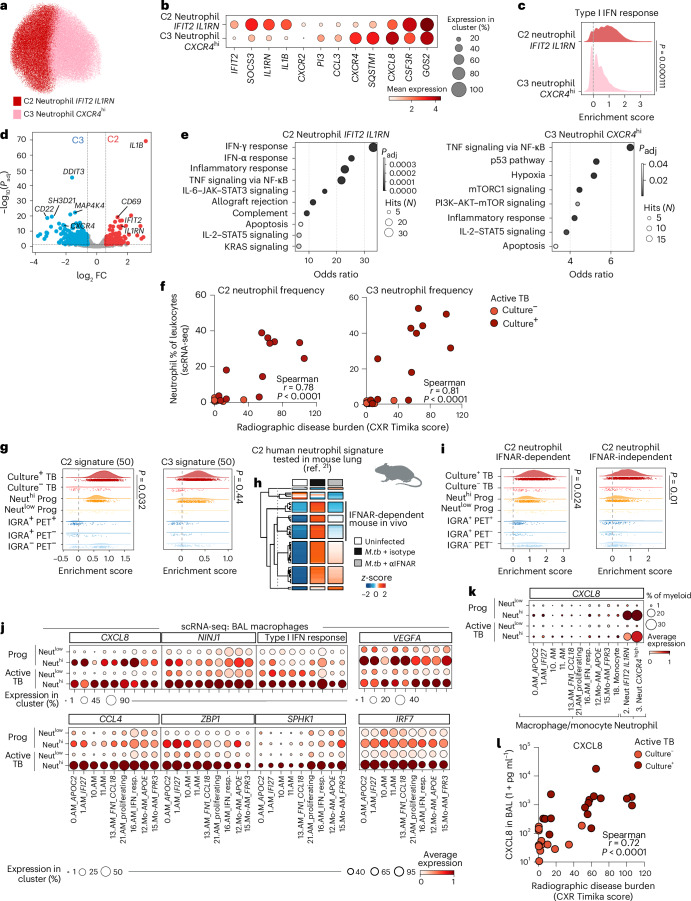
Neutrophil and inflammatory airway macrophage states associated with TB progression and severity. **a**, UMAP of neutrophil scRNA-seq clusters, C2 and C3. **b**, Distinct and shared marker genes in neutrophil clusters. **c**, Distributions of enrichment scores of a type I IFN response signature (defined in Extended Data Fig. 4b). Statistics: Wilcoxon matched-pairs signed-rank test (two tailed) comparing mean enrichment scores per sample between C2 and C3 neutrophils from all participants with ≥20 cells in both clusters (*N* = 42). **d**, Volcano plot of DEGs between neutrophil clusters across all samples (*N* = 42; Wald test with Benjamini–Hochberg multiple testing correction). **e**, Overrepresented pathways within upregulated genes in neutrophil clusters, one-sided Fisher’s exact (hypergeometric) test with Benjamini–Hochberg multiple testing correction. **f**, Abundance of C2 and C3 neutrophils within BAL leukocytes of patients with TB plotted against chest X-ray Timika score. **g**, Distributions of enrichment scores for the top 50 DEGs (defined in Extended Data Fig. 4a) in C2 and C3 neutrophils across total neutrophils in each group. Dots represent individual cells. **h**, Top 50 C2 neutrophil genes were examined by *k*-means clustering in lung bulk RNA-seq data from *M. tuberculosis-*infected mice treated with either anti-IFNAR blocking antibody (αIFNAR) or isotype control. Gene clusters decreased in expression in the lungs of αIFNAR-treated mice are highlighted. Data averaged from three to four mice per group. *M.tb*, *M. tuberculosis*. **i**, Plots as in **g** showing C2 neutrophil genes identified as IFNAR-dependent and -independent in the mouse lung. Group sizes for human scRNA-seq analysis: IGRA^−^ PET–CT^−^(*N* = 9), IGRA^+^PET–CT^−^ (*N* = 5), IGRA^+^PET–CT^+^ (*N* = 6), neutrophil-low progressors (*N* = 3); neutrophil-high progressors (*N* = 3) and patients with BAL culture-negative (*N* = 9) and -positive (*N* = 12) TB. Statistics in **g** and **i**: Mann–Whitney test (two tailed) comparing mean enrichment scores per sample between BAL culture-positive TB (*N* = 12) and neutrophil-high progressors (*N* = 3. **j**,**k**, Expression of indicated genes or signatures from neutrophil-high compared with neutrophil-low patients with active TB and progressors in airway macrophages (**j**) or airway macrophages, monocytes and neutrophils (**k**). Averaged data from all cells from all samples per group are shown in **g** and **i**–**k**. **l**, BAL CXCL8 concentration plotted against chest X-ray Timika score. Results of Spearman correlation analysis (two tailed) shown in **f** and **l**. Mouse icon in **h** created in BioRender; Branchett, W. https://biorender.com/4npgcb4 (2026).

**Extended Data Fig. 4:**
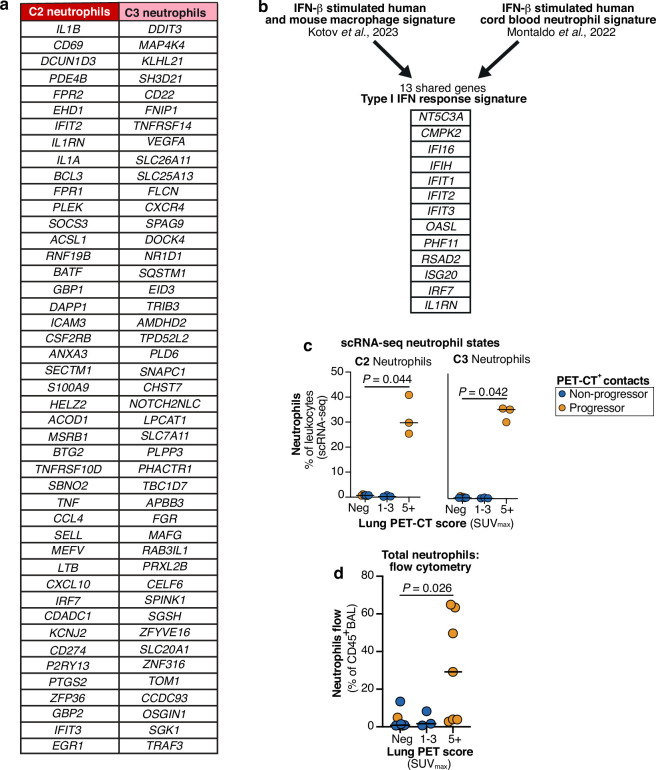
Analysis of airway neutrophils by scRNA-seq and flow cytometry. **a**, Lists of top 50 differentially expressed genes between cluster 2 (C2) and C3 neutrophils, ranked by adjusted *P* value. **b**, Schematic showing selection of a 13 gene myeloid type I IFN response signature by identification of shared genes from the signatures from the indicated publications. **c**, Frequency of C2 and C3 neutrophils as a percentage of total BAL leukocytes by scRNA-seq in all PET-CT^+^ TB contacts, binned by lung PET-CT status and colored by progressor status (N = 3 to 6 per lung PET-CT bin). **d**, Percentage of total neutrophils by flow cytometry as a percentage of total live CD45^+^ BAL leukocytes as determined by flow cytometry, plotted as for (**c**) (N = 3 to 7 per lung PET-CT bin). Points in (**c-d**) represent individual contacts with lines at the median. Statistics: Kruskal-Wallis test with Dunn’s post-hoc test (two-tailed).

The two neutrophil clusters identified by scRNA-seq appeared to represent distinct inflammatory states. While cluster 3 (C3) was distinguished by the marker gene *CXCR4* and higher expression of *DDIT3* and the chemokines *CCL3* and *CXCL8*, cluster 2 (C2) was marked by high expression of *IL1B* and *IL1RN*, encoding IL-1RA (Fig. 4a,b and Extended Data Fig. 4a) and type I IFN-inducible gene signatures (Fig. 4c–e and Extended Data Fig. 4b). Despite the presence of a strong type I IFN-inducible airway neutrophil signature, IL-1β and IL-1RA proteins were also elevated in the BAL of patients with culture-positive TB (Extended Data Fig. 3c,d), in whom neutrophils were generally high (Figs. 2b and 3 and Extended Data Fig. 3b).

The frequency of both neutrophil clusters C2 and C3 within BAL leukocytes correlated with radiographic TB disease burden (Fig. 4f). Both scRNA-seq clusters, as well as total neutrophil frequency by flow cytometry, were also elevated in progressing contacts with a high lung PET–CT signal (Extended Data Fig. 4c,d). However, while the C3 neutrophil signature genes were comparably enriched in neutrophils from progressors and patients with TB, C2 neutrophil genes (Extended Data Fig. 4a) showed significantly greater enrichment in active TB compared with progressors (Fig. 4g), suggesting that this highly inflammatory neutrophil signature increases with more advanced disease. Leveraging our published RNA-seq data of IFNαβ receptor (IFNAR) blockade in a severe mouse TB model^17,21^, the human C2 neutrophil signature genes were found to segregate into clusters with IFNAR-dependent (for example, *IFIT2* and *IFIT3*) and -independent (for example, *ICAM1* and *CD274*) expression in mouse lung during *M. tuberculosis* infection (Fig. 4h). Both component gene sets of the C2 signature showed significantly greater enrichment in TB compared with progressors (Fig. 4i), suggesting that both type I IFN-dependent and -independent activation signatures of airway neutrophils increase with more advanced TB disease.

### Neutrophil accumulation in patients with TB and progressors is accompanied by widespread inflammatory changes in airway macrophages

**Extended Data Fig. 5:**
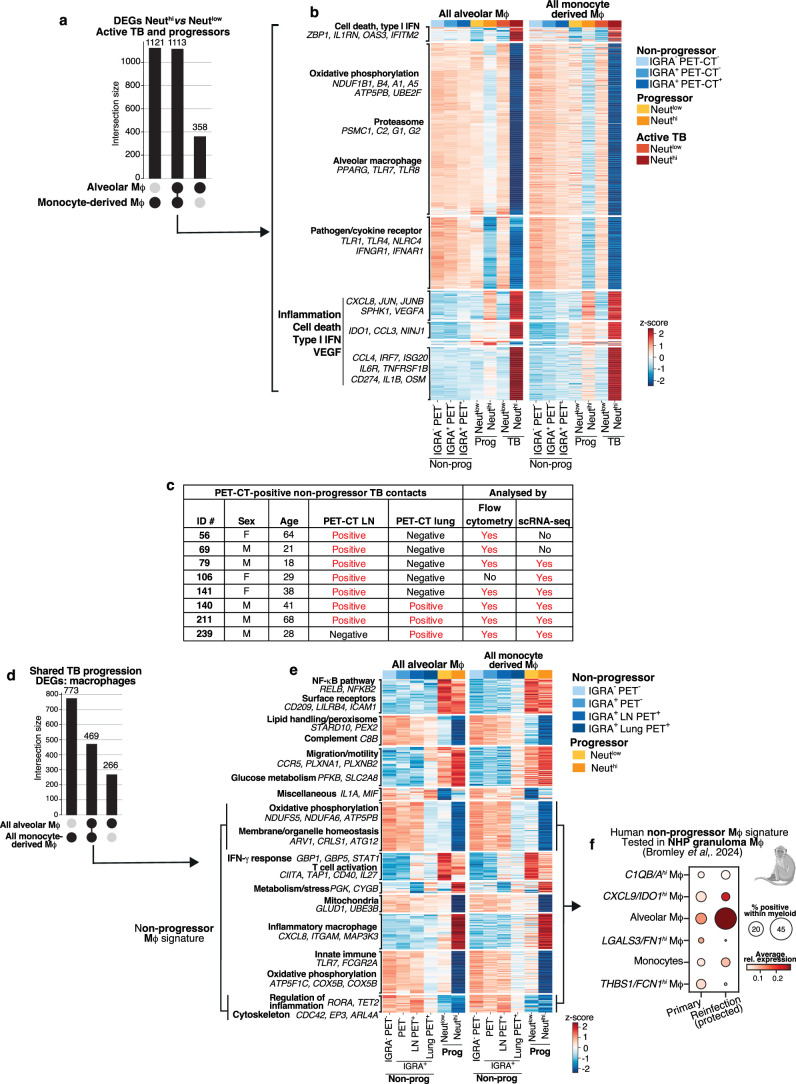
Transcriptional changes in human airway macrophages with TB progression. **a**, Upset plot showing shared and unique differentially expressed gene numbers between alveolar macrophage and monocyte-derived macrophage analyses comparing Neutrophil-high (N = 11) and -low (N = 16) TB patients and progressors. **b**, Heatmap showing k-means clustering of shared differentially expressed genes from **a** across groups IGRA^−^PET-CT^−^ (N = 9), IGRA^+^ PET-CT^−^ (N = 5), IGRA^+^ LN PET-CT^+^ (N = 6), IGRA^+^ lung PET-CT^+^ (N = 3), neutrophil-low progressors (N = 3), neutrophil-high progressors (N = 3), neutrophil-low (N = 13) and neutrophil-high (N = 8) TB patients. **c**, Table showing details of intrathoracic LN and lung parenchyma PET-CT status in PET-CT^+^ IGRA^+^ non-progressors, as used to sub-group non-progressors for differential expression analysis. LN PET-CT^+^ IGRA^+^ non-progressors were defined as those with positive LN PET-CT signal, without positive lung PET-CT signal. **d**, Upset plot showing shared and distinct differentially expressed genes in alveolar and monocyte-derived macrophage scRNA-seq clusters combined from comparisons between progressor and non-progressor sub-groups (detailed in Supplementary Table 7). **e**, Heatmap showing k-means clustering of shared alveolar and monocyte-derived macrophage differentially expressed genes across groups. **f**, Expression of a 50 gene signature of human airway macrophages that decreased in TB progressors, shown across macrophage scRNA-seq clusters from NHP granulomas in primary (N = 10) *M. tuberculosis* infection compared to reinfection (N = 8). NHP icon in **f** created in BioRender; Branchett, W. https://biorender.com/0psmrlu (2026).

To more broadly examine the airway inflammatory landscape accompanying elevated BAL neutrophils in human TB and progressors, we compared gene expression in airway macrophages between neutrophil-high and neutrophil-low patients with TB and progressors (Extended Data Fig. 5a,b), with neutrophil-high defined hereafter as those with ≥15% neutrophils within BAL CD45^+^ leukocytes by flow cytometry. Neutrophil-high patients and progressors exhibited increased expression of genes enriched for functions such as chemotaxis, inflammatory cell death and VEGF signaling across all BAL macrophage populations, including resident-like AMs and monocyte-derived macrophages (Fig. 4j and Extended Data Fig. 5a,b). BAL macrophages in the neutrophil-high state also displayed elevated type I IFN-inducible gene expression, which was more pronounced in active TB than progressors (Fig. 4j), mirroring results in neutrophils (Fig. 4g,i). *CXCL8* expression was also broadly increased across airway macrophage subsets in the neutrophil-high state (Fig. 4j), suggesting that macrophages may contribute to continued neutrophil recruitment, although *CXCL8*-expressing neutrophils far outnumbered macrophages in these patients (Fig. 4k). Together, these findings are consistent with airway neutrophil accumulation marking a wider pro-inflammatory state across resident and monocyte-derived airway macrophages. CXCL8 protein concentration in BAL supernatants strongly correlated with radiographic TB disease burden, consistent with the neutrophil-dominated airway inflammatory state accompanying more severe disease (Fig. 4l).

### Human TB airway inflammatory neutrophil and macrophage signatures are recapitulated in lung tissue of NHP and mice with TB disease

**Fig. 5:**
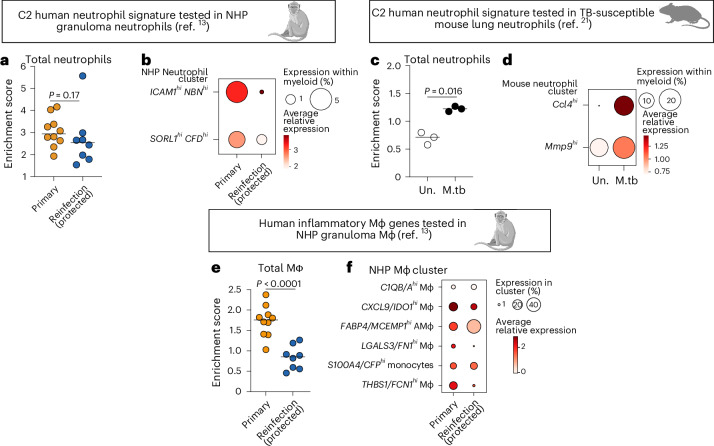
Airway inflammatory myeloid signatures from human patients with TB and progressors are enriched in lungs from animal models of TB. **a**, Mean enrichment scores of the top 50 C2 neutrophil genes, as defined in Extended Data Fig. 4a, shown in total neutrophils from NHP granulomas in primary (*N* = 10) *M. tuberculosis* infection compared with reinfection (protected) (*N* = 8). Points show data from individual granulomas with lines at the median. Statistics: Mann–Whitney test (two tailed). **b**, Relative expression of C2 neutrophil genes in the two published NHP neutrophil clusters. **c**, Mean enrichment scores of the top 50 C2 neutrophil genes shown in total neutrophils per mouse from lungs of uninfected (Un., *N* = 3) mice and at day 20 after infection with *M. tuberculosis* (*Mtb* *N* = 3). Statistics: unpaired *t*-test (two tailed). *M.tb*, *M. tuberculosis*; Un., uninfected. **d**, Relative expression of C2 neutrophil genes in mouse TB neutrophil clusters. **e**, Mean enrichment scores of a 50-gene signature of human airway inflammatory macrophages (Mφ; see the Methods and Extended Data Fig. 5a,b) shown in total macrophages from NHP granulomas in primary (*N* = 10) *M. tuberculosis* infection compared with reinfection (protected) (*N* = 8). Points show data from individual granulomas with lines at the mean. Statistics: unpaired *t*-test (two tailed). **f**, Average expression of the 50-gene human macrophage signature as in (**e**), shown across the different NHP granuloma macrophage clusters. Data are averaged from all granuloma samples per group as shown in (**e**). Icons created in BioRender; NHP, Branchett, W. https://biorender.com/0psmrlu (2026); mouse, Branchett, W. https://biorender.com/4npgcb4 (2026).

To examine our human TB disease-associated immune signatures directly at the site of *M. tuberculosis* infection and determine their association with disease control, we analyzed published scRNA-seq data from lung granulomas of nonhuman primates (NHP), during either primary TB disease or a secondary *M. tuberculosis* infection where CD4^+^ T cell-dependent protection had been achieved through a previous antibiotic-treated infection^13^. Our human C2 neutrophil signature was found to be highly enriched in neutrophils from the NHP granuloma during primary infection (Fig. 5a). The two granuloma neutrophil subclusters identified in the NHP study both expressed this C2 signature during the primary NHP infection but were much less abundant in the granulomas of protected reinfected animals (Fig. 5b). This human C2 signature was also enriched in two lung neutrophil clusters in infected TB-susceptible mice, where pathogenesis is dependent on highly activated neutrophils^17,21^ (Fig. 5c,d). A combined human airway macrophage transcriptional signature of failed immunity in our neutrophil-high patients with TB and progressors was highly enriched in granuloma macrophages of *M. tuberculosis*-infected NHP, most strikingly in disease-associated *CXCL9*/*IDO1*^hi^ macrophages, and diminished in the context of protective immunity from previous infection^13^ (Fig. 5e,f). Conversely, we observed reduced expression of genes related to core AM functions such as lipid metabolism, oxidative phosphorylation and organelle homeostasis in airway macrophages of contacts progressing to TB (Extended Data Fig. 5c–e). A metabolic shift from oxidative phosphorylation and fatty acid oxidation toward glycolysis in macrophages as *M. tuberculosis* infection progresses has been reported in mice, with highly glycolytic macrophages having superior antimycobacterial capacity but probably also greater potential to cause tissue damage^23,24^. This human nonprogressor airway macrophage gene signature was found to be enriched in AM-like cells present in granulomas of immune-protected NHP^13^ (Extended Data Fig. 5f). Our transcriptomic analysis of human airways therefore reveals inflammatory neutrophil and macrophage signatures of TB disease that are consistent across species.

### Dysfunctional T cell states in patients with TB and progressing contacts exhibiting high airway neutrophils

**Fig. 6:**
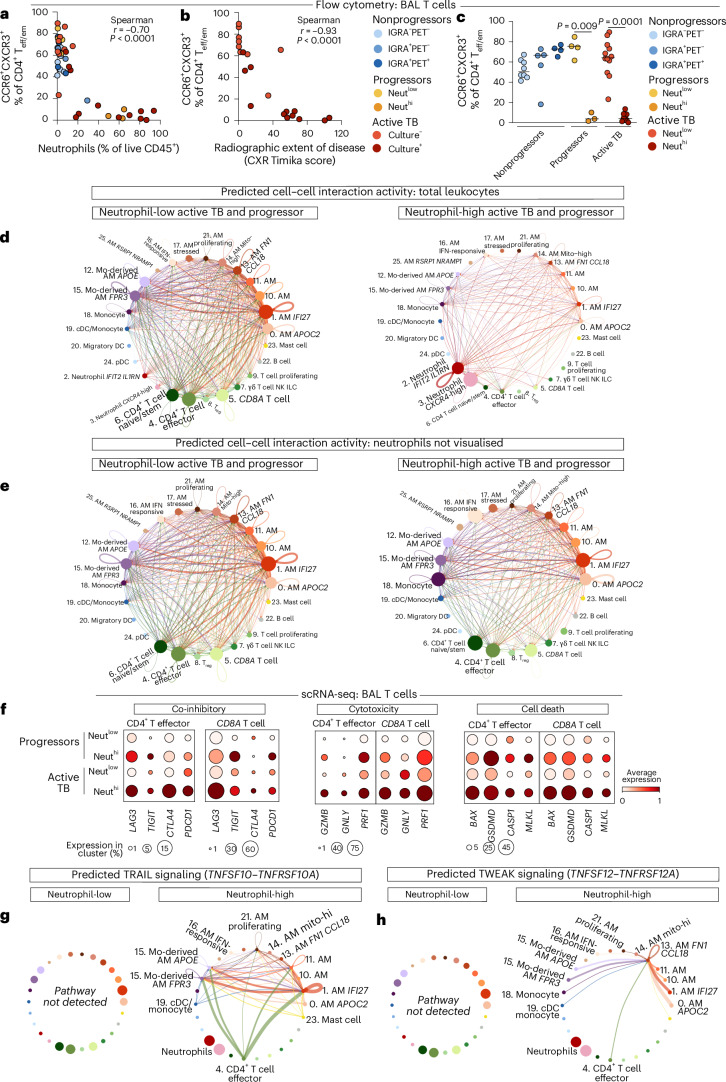
Neutrophil accumulation in patients with TB and progressing contacts is accompanied by signatures of dysfunction in airway T cells. **a**,**b**, Frequencies of CCR6^+^CXCR3^+^CD4^+^ T_eff/em_ cells within total CD4 T_eff/em_, as determined by flow cytometry in IGRA^−^ PET–CT^−^(*N* = 8), IGRA^+^PET–CT^−^ (*N* = 5), IGRA^+^PET–CT^+^ (*N* = 4), neutrophil-low progressors (*N* = 4), neutrophil-high progressors (*N* = 3) and TB BAL culture-negative (*N* = 9) and -positive (*N* = 12) patients with TB. Data are plotted against the proportion of neutrophils in BAL (**a**) and chest X-ray Timika scores (**b**). Results of Spearman correlation analysis (two tailed) are shown. **c**, Flow cytometry as in **a** and **b** shown across study groups, with patients with active TB split into neutrophil-low (*N* = 12) and -high (*N* = 9) subgroups. Points show individual participants with lines at the median. Statistics: Kruskal–Wallis test with Dunn’s post hoc test (two tailed). **d**, CellChat-inferred receptor–ligand interactions analysis comparing neutrophil-high (*N* = 11) to neutrophil-low (*N* = 16) patients with TB and progressors, showing predicted total interaction strength between scRNA-seq clusters in each condition. pDC, plasmacytoid dendritic cell. **e**, The same CellChat-inferred receptor–ligand interactions as in **d**, this time shown with neutrophils excluded from the visualization to allow visualization of T cell–myeloid interactions. **f**, Expression of representative genes increased in airway T cells from neutrophil-high compared with neutrophil-low patients with active TB and progressors. Data shown are averaged from all cells from all samples per group: neutrophil-low progressors (*N* = 3), neutrophil-high progressors (*N* = 3) and neutrophil-low (*N* = 13) and neutrophil-high (*N* = 8) patients with TB. **g**,**h**, CellChat analysis showing predicted activity of TRAIL and TWEAK pathways between leukocyte populations in BAL. In **d**, **e**, **g** and **h**, circle sizes are proportional to cluster abundance, lines are colored according to the ligand-expressing population and line thickness represents the relative predicted strength of interactions.

**Extended Data Fig. 6:**
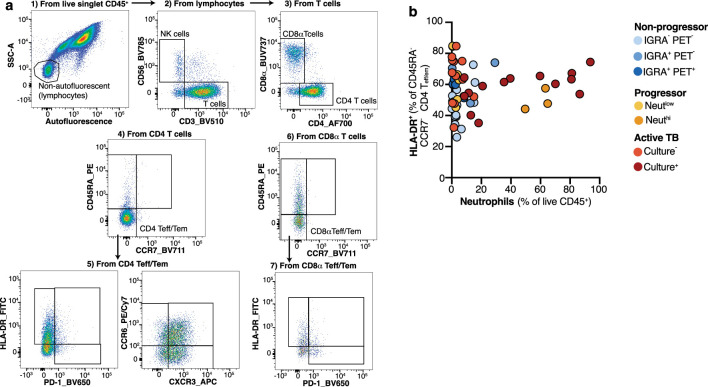
Airway T cell flow cytometry and association with airway neutrophil frequency. **a**, Flow cytometry gating strategy for the T cell focused panel, used to identify different T cell phenotypic and activation markers. **b**, HLA-DR^+^ CD4^+^ CD45RA^−^ CCR7^−^ T_eff/em_ as a percentage of total CD4^+^ CD45RA^−^CCR7^−^ T_eff/em_, plotted against neutrophil frequency in BAL by flow cytometry. Data are shown from IGRA^−^ PET-CT^−^(N = 15), IGRA^+^ PET-CT^−^ (N = 6), IGRA^+^ PET-CT^+^ (N = 7), neutrophil-low progressors (N = 6), neutrophil-high progressors (N = 3), BAL culture-negative TB patients (N = 12) and BAL culture-positive TB patients (N = 14).

To understand the potential impact of neutrophils on airway T cell responses in human TB, we assessed T cell phenotype by flow cytometry in patients with TB and contacts (Extended Data Fig. 6a). Strikingly, surface co-expression of chemokine receptors CXCR3 and CCR6 by CD45RA^−^CCR7^−^CD4^+^ effector/effector memory (T_eff/em_) cells was almost completely absent in neutrophil-high patients with TB and progressors (Fig. 6a,c). CXCR3^+^CCR6^+^CD4^+^ T_eff/em_ frequency inversely correlated with radiographic TB disease burden (Fig. 6b), suggesting their role in infection control, consistent with reports that CXCR3^+^CCR6^+^ marks a putative protective phenotype of *M. tuberculosis* antigen-specific CD4^+^ T cells in human blood^25^ and NHP BAL^26^ in latent TB. In contrast to co-expression of CXCR3 and CCR6, expression of the activation marker HLA-DR on CD4 T_eff/em_ cells showed no correlation with the extent of neutrophilic airway inflammation (Extended Data Fig. 6b).

**Extended Data Fig. 7:**
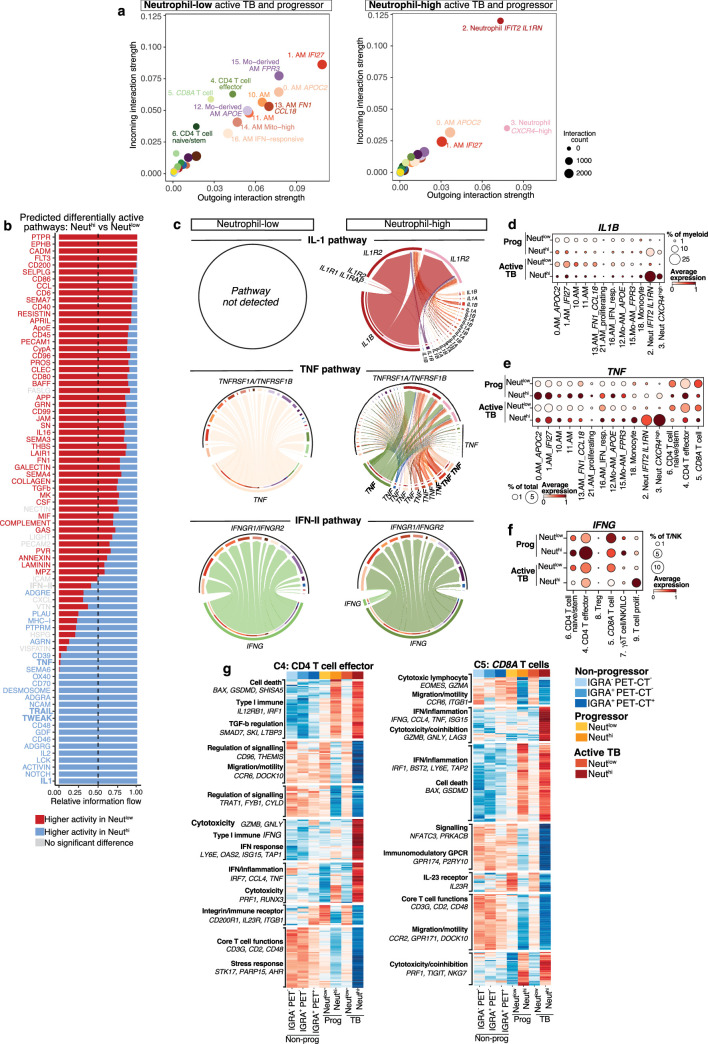
Predicted immune interactions and T cell signatures in airways of neutrophil-low and -high TB patients and progressors. **a**, Predicted outgoing and incoming signal strength per scRNA-seq cluster as determined by CellChat in neutrophil-low (N = 16) and neutrophil-high (N = 11) TB patients and progressors. **b**, Bar plot showing predicted differentially active pathways determined by permutation analysis in CellChat, with *P* < 0.05 taken as significant. **c**, Plots showing the predicted signaling between the indicated scRNA-Seq clusters and receptor-ligand pairs in each condition. Data shown are averaged from all samples per group. **d-f**, Dot plots showing expression of key ligand genes across relevant scRNA-seq clusters in neutrophil-low progressors (N = 3), neutrophil-high progressors (N = 3); neutrophil-low TB patients (N = 13) and neutrophil-high TB patients (N = 8). **g**, Heatmaps showing k-means clustering of differentially expressed genes from the indicated T cell scRNA-seq clusters from neutrophil-low and -high TB patients and progressors, shown across these groups, as well as IGRA^−^PET-CT^−^(N = 9), IGRA^+^ PET-CT^−^ (N = 5) and IGRA^+^ PET-CT^+^ (N = 6) non-progressors.

To more broadly examine the potential effect of high airway neutrophils on T cell responses, we compared quantitative and qualitative changes at single-cell level between neutrophil-high and neutrophil-low subgroups of patients with TB and progressors. Bioinformatic receptor–ligand analysis predicted extensive macrophage–macrophage and T cell–macrophage interactions in neutrophil-low patients with TB and progressors, which were not apparent in the neutrophil-high state, where neutrophils dominated the predicted sending and receiving of signals (Fig. 6d and Extended Data Fig. 7a). Removal of the dominant neutrophil-dependent interactions from the visualization revealed the presence of underlying T cell–macrophage and macrophage–macrophage interactions but at a much reduced level than in the neutrophil-low state (Fig. 6e). This suggests that neutrophils may inhibit, as well as obscure, T cell–macrophage interactions, supporting observations in TB-susceptible mouse models of excessive neutrophil recruitment opposing protective T cell responses^21,22^.

IL-1β and TNF are well-established protective cytokines against *M. tuberculosis* infection^18^; however, increased IL-1 and TNF pathway activity was predicted in neutrophil-high compared with neutrophil-low patients and progressors (Extended Data Fig. 7b–e), probably reflecting the more inflammatory airway immune environment in these participants at this established disease stage. Notably, IL-1 pathway activity was largely predicted to be to the nonsignaling decoy receptor, encoded by *IL1R2*, on neutrophils themselves (Extended Data Fig. 7c). Predicted IFN-γ signaling was comparable between the neutrophil-high and -low groups (Extended Data Fig. 7c), although IFN-γ protein was most elevated in BAL fluid from patients with culture-positive TB (Extended Data Fig. 3c). IFN-γ is a key protective cytokine during mycobacterial infection, and T-cell-derived IFN-γ has been reported to limit neutrophilic lung pathology in a mouse TB model^27^. However, vaccine studies have suggested that IFN-γ alone is insufficient as a correlate of protection against TB^5^, while experimental TB model studies have indicated that excessive IFN-γ can contribute to immunopathology^28^. Elevated BAL IFN-γ in patients with culture-positive TB in our study therefore probably reflects greater inflammation and immune activation in the airways of these patients. Notably, diminished expression of *IFNG* was observed in the CD4^+^ T effector and *CD8A*^+^ T cell clusters of neutrophil-high patients with TB, suggesting that cellular sources of IFN-γ may differ with moderate or severe disease and influence outcome (Extended Data Fig. 7f).

Compared with neutrophil-low groups, higher levels of coinhibitory receptors, cytotoxic mediators and genes related to apoptotic and inflammatory cell death were observed in CD4^+^ T effector and *CD8A*^+^ T cell clusters from neutrophil-high patients with TB and progressors (Fig. 6f and Extended Data Fig. 7g). Moreover, the TRAIL and TWEAK pathways of extrinsic apoptosis were predicted to be active in neutrophil-high but not neutrophil-low patient groups, in part driven by expression of the ligands by CD4^+^ T cells (Fig. 6g,h). Collectively these data suggest that a dysregulated inflammatory response, together with chronic activation, exhaustion and cell death in airway T cells probably contribute to failed immune control of *M. tuberculosis* accompanying excessive neutrophil accumulation.

### Cross-species T cell signatures of *M. tuberculosis* control revealed in airways of human TB contacts

**Fig. 7:**
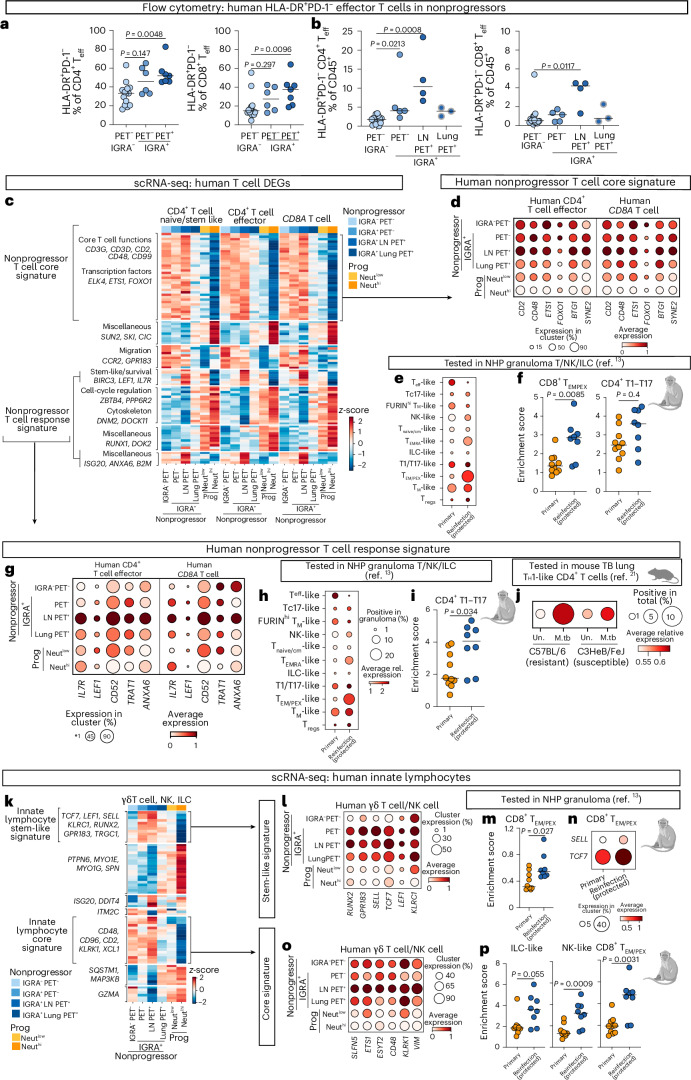
Cross-species lymphocyte signatures of pulmonary *M. tuberculosis* infection control. **a**,**b**, Flow cytometry data from BAL of nonprogressor contacts showing HLA-DR^+^PD-1^−^ CD4^+^ and CD8^+^ T_eff/em_ cells either as percentages of the parent T_eff/em_ population (**a**) or total live CD45^+^ leukocytes (**b**). Points show individual contacts with lines at the median: IGRA^−^PET–CT^−^(*N* = 15), IGRA^+^PET^−^CT^−^ (*N* = 5), IGRA^+^ LN PET–CT^+^ (*N* = 4), IGRA^+^ lung PET–CT^+^ (*N* = 3). Statistics: Kruskal–Wallis test with Dunn’s post hoc test (two tailed). **c,k**, Heat maps showing *k*-means clustering of shared DEGs from across major scRNA-seq T cell clusters (**c**) or the γδT cell, NK and ILC scRNA-seq cluster (**k**), combined from comparisons between progressor and nonprogressor subgroups (detailed in Supplementary Table 7); IGRA^−^PET–CT^−^(*N* = 9), IGRA^+^PET^−^CT^−^ (*N* = 5), IGRA^+^ LN PET–CT^+^ (*N* = 3), IGRA^+^ lung PET–CT^+^ (*N* = 3), neutrophil-low progressors (*N* = 3) and neutrophil-high progressors (*N* = 3). **d**,**g**,**I**,**o**, Expression of representative genes from the indicated nonprogressor-associated lymphocyte signatures: T cell core (**d**), T cell response (**g**), γδT cell/NK cell stem like (**l**) and γδT cell/NK cell core (**o**) across TB contact groups, averaged across all samples per group. **e**,**f**,**h**,**i**,**m**,**n**,**p**, The indicated human BAL nonprogressor lymphocyte signatures: T cell core (**e**,**f**), T cell response (**h**,**i**), γδT cell/NK cell stem like (**m**,**n**) and γδT cell/NK cell core (**p**) were examined in lung granuloma T/NK cell scRNA-seq data from NHP and compared between those from primary *M. tuberculosis* infection (*N* = 10) and in the context of immune protection during reinfection (*N* = 8). Data are shown as either mean enrichment scores per granuloma, with lines at the median (**f**,**i**,**m**,**p**; statistics: two-tailed Mann–Whitney *t*-test) or average expression across the indicated NHP granuloma scRNA-seq clusters (**e**,**h**,**n**). **j**, Average expression of the human BAL nonprogressor T cell response signature in T_H_1-like CD4^+^ T cells in TB-resistant or susceptible mice (C57BL/6 or C3HeB/FeJ, respectively) lungs in uninfected (un.) mice and at 20 days after infection (*Mtb*). Averaged data from *N* = 3 mice per group are shown. Icons created in BioRender; NHP, Branchett, W. https://biorender.com/0psmrlu (2026); mouse, Branchett, W. https://biorender.com/4npgcb4 (2026).

**Extended Data Fig. 8:**
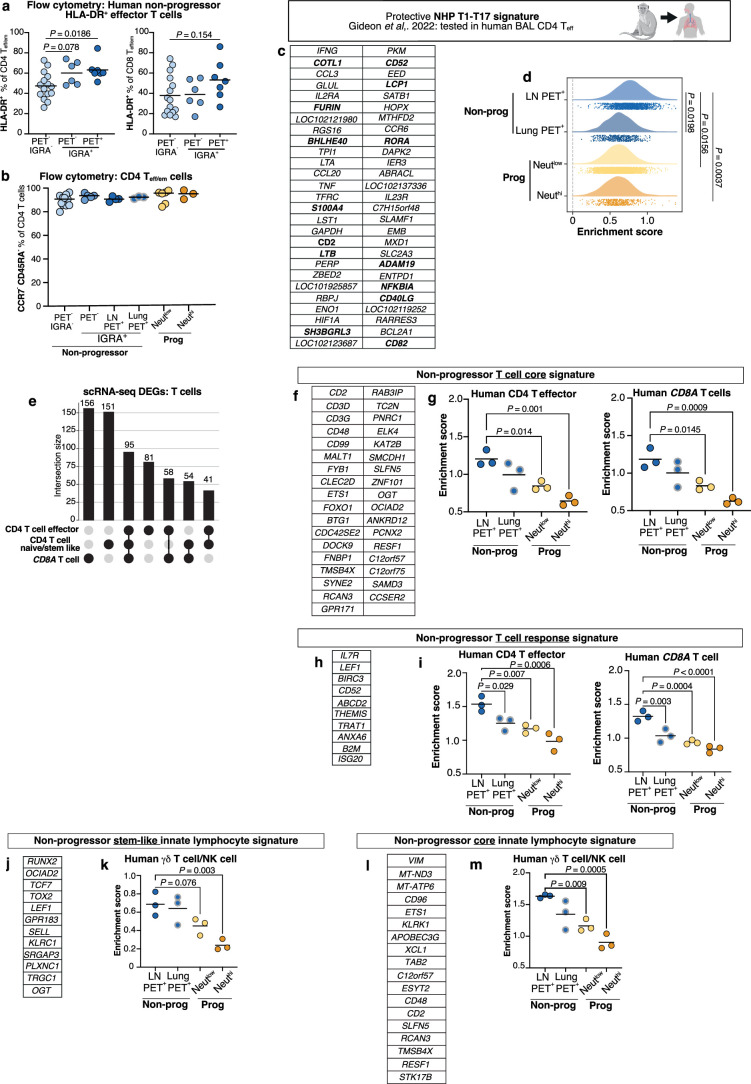
Lymphocyte signatures of non-progression in human TB contacts. **a-b**, T cell phenotype as determined by flow cytometry in IGRA^−^ PET-CT^−^(N = 15), IGRA^+^ PET-CT^−^ (N = 6), IGRA^+^ PET-CT^+^ (N = 7), neutrophil-low progressor (N = 5) and neutrophil-high (N = 3) progressor BAL. **a**, Percentage of T_eff/em_ CD4 and CD8 T cells expressing HLA-DR. Points show data from individual contacts with lines at the median. Statistics: Kruskal-Wallis test with Dunn’s *post hoc* test (two-tailed). **b**, Percentage of total CD4 T cells with a CD45RA^−^ CCR7^−^ T_eff/em_ surface phenotype. Points show data from individual participants with lines at the mean. **c-d**, List and enrichment scores of top 50 signature genes of the T1-T17 T cell population associated with protection in NHP granulomas, in the CD4 T effector cluster from human BAL. Combined data from N = 3 individuals per group are shown. Dots under density plots represent individual cells. Statistics: Kruskal-Wallis test with Dunn’s *post hoc* test (two-tailed) comparing mean enrichment scores from each participant (N = 3). Genes in bold in (**c**) are those contributing ≥1% to the observed enrichment in our human BAL CD4 T effector cells. **e**, Upset plot showing shared and distinct differentially expressed genes in the major T cell scRNA-seq clusters combined from comparisons between progressor and non-progressor sub-groups (detailed in Supplementary Table 7); IGRA^−^PET-CT^−^(N = 9), IGRA^+^ PET-CT^−^ (N = 5), IGRA^+^ LN PET-CT^+^ (N = 3), IGRA^+^ lung PET-CT^+^ (N = 3), neutrophil-low progressors (N = 3) and neutrophil-high progressors (N = 3). **f, h, j, l**, Lists of genes within the indicated non-progressor lymphocyte signatures as shown in Fig. 7. **g, i, k, m**, Mean enrichment scores of the indicated signatures in the indicated scRNA-seq populations in individual contacts. Points show data from individual participants with lines at the mean (N = 3 per group). Statistics: one-way ANOVA with Sidak’s post-hoc test (two-tailed). Icons in **c** created in BioRender; NHP: Branchett, W. https://biorender.com/0psmrlu (2026); Human: Branchett, W. https://biorender.com/uhc5llr (2026).

We further investigated our datasets for evidence of active protective immune responses in contacts who did not progress to TB. Reasoning that IGRA^+^ nonprogressors with a positive thoracic PET–CT represent those mounting T-cell-dependent responses to current *M. tuberculosis* infection, we compared effector T cells in this group with the other nonprogressors in our study. Flow cytometric analysis revealed that the frequency of HLA-DR^+^PD-1^−^ BAL T_eff/em_ was increased in IGRA^+^PET–CT^+^ as compared with IGRA^−^PET–CT^−^ nonprogressors (Fig. 7a), to a greater extent than seen for total HLA–DR^+^ T_eff/em_ (Extended Data Fig. 8a). Abundance of these T cells was greatest in those IGRA^+^ nonprogressors with a positive thoracic lymph node (LN), but not lung, PET–CT signal (Fig. 7b and Extended Data Fig. 5c). These data suggest that a PET–CT signal in the LN of nonprogressors may be associated with a protective airway T cell response. In support of this, we found that a published protective T1–T17 signature of *M. tuberculosis* control in NHP granulomas^15^ was enriched in our scRNA-seq CD4^+^ T-effector population from human BAL of IGRA^+^ LN PET–CT^+^ nonprogressors (Extended Data Fig. 8c,d). To further explore potentially protective airway CD4^+^ and CD8^+^ T cell responses between progressor and nonprogressor subgroups now also including their LN and lung PET–CT status, we performed differential expression analysis in our human scRNA-seq dataset, identifying common patterns of differentially expressed genes (DEGs) among the major T cell clusters (Fig. 7c and Extended Data Fig. 8e).

A nonprogressor T cell core signature was found to be expressed in IGRA^−^, IGRA^+^PET–CT^−^ and IGRA^+^ LN PET–CT^+^ nonprogressors but reduced in lung PET–CT^+^ nonprogressors, suggesting a gene expression program that is lost upon lung inflammation (Fig. 7c and Extended Data Fig. 8f,g). This T cell core signature was also diminished as contacts progressed to TB (progressors). This signature included cell–cell adhesion genes *CD2* and *CD48*, the transcriptional regulator *ETS1* and the T cell quiescence factors *FOXO1*^29^ and *BTG1*^30^ (Fig. 7c,d), suggestive of a restrained activation state that was diminished upon increased lung inflammation and/or upon progression to TB. Expression of these quiescence genes was observed in the *CD8A*^+^ T cell and CD4^+^ T effector clusters and not restricted to the CD4^+^ naive/stem-like cluster (Fig. 7c,d) and was not accompanied by a change in frequency of CD45RA^−^CCR7^−^ T_eff/em_ airway CD4^+^ T cells across groups (Extended Data Fig. 8b). We determined that our human nonprogressor T cell core signature was evident in a published population of *GZMK*^hi^ CD8^+^ T effector memory/precursor exhausted (T_EM/PEX_)-like T cells identified by scRNA-seq in granulomas from NHP, which was more abundant in the context of immune protection^13^ (Fig. 7e,f).

A second T cell signature was strikingly increased in the LN PET–CT^+^ IGRA^+^ nonprogressor group only and not in the other nonprogressor groups, suggestive of a recent protective immune response to *M. tuberculosis* infection, rather than a cleared or past infection (Fig. 7c and Extended Data Fig. 8h,i). This nonprogressor T cell response signature included *IL7R* and *LEF1*, both associated with a stem-like T cell state^31,32^. Accompanying this stem-like profile was expression of genes implicated in regulation of T cell receptor signaling and membrane dynamics, *CD52*^33,34^, *TRAT1*^35^ and *ANXA6*^36^ (Fig. 7c,g). This human airway nonprogressor stem-like T cell response signature was increased in granuloma T cell populations identified by scRNA-seq from immune-protected NHP as compared with those with primary infection and disease, including the CD4^+^ T1–T17 effector cluster^13^ (Fig. 7h,i). We additionally observed this human T cell response signature in T_H_1-like CD4^+^ T cells in lungs of relatively TB-resistant C57BL/6 mice early in infection, at a higher frequency than in TB-susceptible C3HeB/FeJ mice that fail to control *M. tuberculosis* infection^21^ (Fig. 7j).

A stem-like innate lymphocyte signature, including *TCF7*, *LEF1* and *SELL*, was enriched in IGRA^+^ but not IGRA^−^ nonprogressors within a small cluster of γδ/NK/innate lymphoid (ILC) cells identified in our scRNA-seq analysis, which was decreased in TB progressors (Fig. 7k,l and Extended Data Fig. 8j,k). A second innate lymphocyte signature, including *KLRK1*, encoding the activating receptor NKG2D, was observed in IGRA^−^, as well as in IGRA^+^PET–CT^−^ and IGRA^+^ LN PET–CT^+^ nonprogressors, but was reduced in lung PET–CT^+^ nonprogressors, again reflecting its potential loss upon lung inflammation (Fig. 7k,o and Extended Data Fig. 8l,m). Supporting a potential protective role for innate lymphocytes in our human nonprogressors, NK cells have been associated with controlled *M. tuberculosis* infection in human blood^14,37^ and NHP lung^14^. Moreover, our human innate-like lymphocyte signatures of nonprogressors showed greater enrichment in NHP granulomas from immune-protected animals, as compared with those from primary infection, including in the CD8 T_EM/PEX_-like population in granulomas that was associated with protection in the published NHP study^13^ (Fig. 7m,n,p).

Collectively, our findings in human airways of TB contacts who controlled infection suggest that a restrained, stem-like activation state in T cells and innate lymphocytes is maintained to ensure durable protection from progression to TB disease.

## Discussion

While most individuals infected with *M. tuberculosis* control the infection and remain asymptomatic, the local early immune factors that dictate protection or disease progression have not been defined. To investigate this, we have charted the airway immune response at single-cell resolution in an extensively characterized and prospectively observed cohort of household TB contacts. Our in-depth analysis has revealed early immune changes at the site of *M. tuberculosis* infection that reflect protection in contacts who remained clinically healthy and radiologically stable by PET–CT and, conversely, inflammatory signatures of failed immunity in individuals who progressed to TB. Using unbiased analysis of bulk and scRNA–seq of BAL samples, we here report an inverse relationship between neutrophils and T cells in patients with TB and contacts that progress to TB, with neutrophilic airway inflammation associated with signatures of exhaustion, cytotoxicity and cell death in T cells. Conversely, T cell signatures of protection in contacts who remained healthy were dominated by genes involved in regulation, quiescence and a stem-like profile. These human airway immune signatures of protection and progression were recapitulated in published data from lung tissue from NHP and mouse TB models with controlled infection or disease, respectively, supporting their functional role in infection outcome. It should be noted that the lower airways and parenchymal granulomas represent distinct tissue compartments and that detection of common immune signatures in human airway samples and in lung lesions from animal models does not necessarily reflect free movement of cells and cytokines between these compartments. However, our findings that signatures from human airways are captured in granuloma scRNA-seq datasets from NHP indicates that BAL samples from patients with TB and their contacts can reveal immune responses also present in the TB granuloma. This is notable, as human lung tissue from patients with TB is typically only obtained postmortem or following resection in the case of poorly controlled disease and cannot be obtained from healthy individuals, such as human TB contacts.

Lung neutrophil accumulation is a common end point of failed immune control of *M. tuberculosis* in mouse models^17,27,38^ and increased neutrophil numbers in BAL are a feature of more advanced active TB in humans^39^. Our current findings suggest that neutrophils may drive TB progression as well as advanced TB disease. In-depth analysis of scRNA-seq data from airway neutrophils revealed two distinct transcriptional states in patients with culture-positive TB and a subset of TB progressors, with both type I IFN-dependent and type I IFN-independent inflammatory signatures associating with more advanced disease. While a blood neutrophil type I IFN-inducible signature in humans has been associated with more advanced TB disease^11,12,18^, IFNAR blockade only partially protected genetically susceptible mice from severe TB^17,21^, supporting the role of type I IFN-independent, as well as -dependent mechanisms of neutrophil-driven TB progression, as suggested by our current human TB contact study. The neutrophil-attractant chemokine CXCL8 was elevated in human BAL and correlated with radiographic TB disease burden consistent with the neutrophil-dominated airway inflammatory state accompanying more severe disease. This is consistent with results in TB-susceptible mouse models, where high expression of the functional CXCL8 homolog, CXCL2, was proposed to drive swarming of neutrophils at the site of *M. tuberculosis* infection^21,38^. Granulocytes present a permissive niche for *M. tuberculosis* in the mouse lung^40^, and neutrophils are the most prevalent *M. tuberculosis*-infected cells in airways of human patients with TB^41^. Recently, a metabolically active subset of lung neutrophils in the *M. tuberculosis*-infected murine lung was shown to exhibit increased lifespan and permissiveness to intracellular survival of *M. tuberculosis*^42^. Whether the different neutrophil transcriptional states identified in our human airway study represent different degrees of permissiveness to *M. tuberculosis* infection and replication remains an open question. Airway neutrophil accumulation in human patients with TB and progressors was accompanied by profound transcriptional and phenotypic changes in airway T cells, suggestive of chronic activation and impaired function, along with widespread inflammatory changes in macrophage populations. These findings in human airways support mechanistic studies in mouse TB models, where neutrophil accumulation opposes protective macrophage–T cell interactions in the lung^21,22^.

Co-expression of type I IFN-inducible genes and *IL1B* by the C2 cluster of neutrophils in the BAL of patients with TB and progressors in our study is notable because, while type I IFN has shown adverse effects in TB^16,17^, IL-1 signaling has been reported to be protective and functionally antagonizes pathogenic type I IFN signaling in a mouse TB model^43^. As our neutrophil cluster C2 also expressed high levels of *IL1RN*, which antagonizes IL-1 signaling, this may represent an IL-1RA-dependent feedback mechanism to control IL-1 function and untoward inflammation that may be functioning aberrantly during chronic infection. Such feedback regulation is further supported by reports that IL-1RA is induced by type I IFN and its deletion in TB-susceptible mice reduces type I IFN-driven TB disease^16^. The expression of both *IL1RN* and the decoy receptor *IL1R2* by the C2 cluster of neutrophils in BAL of patients with TB and progressors in our current study, together with type I IFN-inducible genes, supports a model in which type I IFN-activated neutrophils contribute to dysfunctional IL-1–IL1R1 signaling in the human TB lung. *Il1b* expression and type I IFN responses have recently been reported to characterize two different hubs within the mouse NeuMap of neutrophil states across tissues and conditions^44^. It is therefore likely that the C2 neutrophil cluster in our analysis, where co-expression of *IL1B* and type I IFN-inducible genes was observed, represents a distinctive and detrimental activation state of airway neutrophils in TB contributing to failed control of this chronic intracellular bacterial infection.

Determinants of an effective protective T cell response to *M. tuberculosis* are not well understood but are likely to depend on temporal and spatial factors, such as the durability of responses and ability of T cells to access and interact with infected macrophages^5,6^. Our findings that T cell signatures of stem-like T cell states, including *LEF1* and *IL7R*, were elevated only in airway T cells from IGRA^+^ LN PET–CT^+^ nonprogressor recent household contacts suggests that it is part of a protective airway response, reflecting current or recent controlled *M. tuberculosis* infection. A role for stem-like T cell states in *M. tuberculosis* control is supported by numerous reports of stem-like memory CD8^+^ T cells providing durable protective memory responses to tumors and chronic viral infections^31,32,45–48^. This is also in line with a proposed protective role of antigen-specific stem-cell memory CD4^+^ T cells in the blood of BCG-vaccinated individuals that correlated with a long-term T cell proliferative response^49^. In addition, a stem-like transcriptional profile has been reported in *M. tuberculosis* antigen-specific CD4^+^ T cells in the blood from individuals who control infection, with *LEF1* and *IL7R* expression specifically increased in IGRA^+^, compared with persistently IGRA^−^ individuals termed resistors^50^. Preservation of a stem-like state in local T cells in IGRA^+^ LN PET–CT^+^ nonprogressors in our study may reflect a more controlled T cell response with capacity to differentiate into protective effector T cells to mediate sustained immune protection as required, in contrast to the terminally differentiated and exhausted T cell signatures observed in neutrophil-high patients with TB and progressors. Our comparative studies demonstrating enrichment of our human BAL T cell signatures of stemness and restrained activation in the context of adaptive immune protection in NHP granuloma T cells^13^ supports an association of these transcriptional states with *M. tuberculosis* control.

Our study of recent human TB household contacts in a low TB-burden setting reveals early immune signatures at the site of *M. tuberculosis* infection associated with infection control or disease progression. Recapitulation of these human signatures in datasets from lungs of experimental animal TB models exhibiting controlled infection or disease strongly supports their involvement in dictating infection outcome. While signatures of disease progression provide targets for further study in the context of host-directed therapy, our stem-like T cell signatures in contacts demonstrating active immune control will be valuable to inform strategies for improved vaccines against TB.

## Methods

### Participant recruitment and clinical study design

This was a 24-month prospective observational cohort study of patients with pulmonary TB and recent household TB contacts, recruited between September 2021 and April 2024 at University Hospitals of Leicester NHS Trust, UK (ISRCTN17985576). The study was approved by the Research and Ethics Committee (REC) for East Midlands–Nottingham 1, Nottingham, UK (REC 21/EM/0139). All patients with TB and household contacts provided informed consent for their participation in the study, including to publication of data arising from this study. Eligible participants were immunocompetent adults aged ≥16 years with suspected TB or recent household contacts of pulmonary TB. Exclusion criteria were significant immunosuppression caused by immunodeficiency disorders or use of T-cell specific or generalized immunosuppressive therapy, including oral corticosteroids at any dose; a history of receiving TB preventive therapy in the preceding 5 years or previous treatment for active TB in the preceding 2 years; and pregnancy or lactation.

A total of 32 adults with pulmonary TB were recruited to the study and underwent BAL sampling. All participants had compatible clinical signs and symptoms and typical imaging features of active TB disease. In 25 participants, microbiological confirmation was achieved by culture and/or Xpert-MTB RIF Ultra testing of either respiratory tract samples (*N* = 22) or from nonrespiratory sites (*N* = 3) in cases with multicompartmental involvement. In seven participants without microbiological confirmation, the diagnosis was supported by histology and/or verified clinical and radiological response to a full course of antitubercular treatment. Two BAL samples from patients with microbiologically confirmed TB were excluded from all analysis owing to excessive blood contamination in one and a subsequent diagnosis of chronic lymphocytic leukemia in the other, leaving 30 samples for analysis. Patients with TB were further grouped on the basis of detection of positive *M. tuberculosis* culture in BAL (Supplementary Table 1b).

Asymptomatic household pulmonary TB contacts were recruited soon after the index case notification. Consenting participants completed a symptom questionnaire and underwent chest radiography (CXR) to exclude active TB. IGRA testing was performed with QuantiFERON-TB GOLD Plus, and among IGRA-positive participants, those that declined TB preventative therapy were retained in the study. All participants were offered bronchoscopy after PET–CT at baseline and after 3 months (median time after the index case notification to recruitment was 23 days). Thereafter, all IGRA-positive participants were actively monitored for evidence of TB progression at 3-monthly intervals with a symptom questionnaire and CXR until 24 months; IGRA-negative participants were passively followed with instructions to contact the study team if any symptoms develop (UK standard of care). At the end of 24 months, participants were contacted to verify that they had remained well. Participants were reimbursed £200 for each research bronchoscopy received, where not required for clinical reasons. A total of 157 contacts were recruited; 4 participants withdrew, 53 declined bronchoscopy and 34 were not offered bronchoscopy (Extended Data Fig. 1a). In total, 66 pulmonary TB contacts provided BAL. Participants were grouped for analysis on the basis of clinical progression, PET–CT features and IGRA results over the study period.

In total, 11 progression events occurred over the follow-up period (Extended Data Fig. 1a,b and Supplementary Table 1c). Progression events were defined by the clinical decision to treat for TB disease and classified into three phenotypes: (1) clinical TB, (2) asymptomatic TB or (3) PET–CT-based progression. Clinical and asymptomatic TB were defined by World Health Organization (WHO) criteria. For clinical TB, this was new symptoms and/or signs of active disease, supported by compatible radiological and/or microbiological and/or histological evidence of TB. For asymptomatic TB, this was CXR and/or microbiological evidence of TB in the absence of any symptoms or signs. In one case (participant no. 75), a progression event was defined by significant interval PET–CT progression in the absence of visible CXR changes that was clinically attributable to TB and that raised sufficient clinical (high risk if left alone) and public health concern (risk of transmission) to arrange invasive investigation. Progression was confirmed by microbiological evidence and compatible histology obtained from the presumed site of disease. BAL was obtained from ten progressors at the point of progression. In one case (no. 222), bronchoscopy was not performed, as progression was characterized by evolving pleural pathology in the absence of any pulmonary involvement. TB in this case was confirmed by pleural biopsy (Supplementary Table 1c and Supplementary Fig. 1).

Demographic details for each study participant are presented in Supplementary Table 1a,b.

### Sample inclusions and exclusions

Current tobacco smokers were excluded from analysis of nonprogressor contact groups (two IGRA^−^PET–CT^−^, three IGRA^+^PET–CT^−^ and three IGRA^+^PET–CT^+^), owing to potential confounding effects of smoking on the subtle immune signatures expected in nonprogressors. A total of 10 of 11 progressors and 25 of 30 patients with active TB were nonsmokers, and no exclusions were made from these groups. Of the remaining 30 IGRA^−^ nonprogressors sampled by bronchoscopy, 11 were excluded from the IGRA^−^ study group for analysis as clinical outliers owing to (1) respiratory symptoms close to the time of sampling attributable to a community-acquired infection, with or without positive BAL bacterial (nonmycobacterial) culture or viral PCR (*N* = 6); (2) without respiratory symptoms but having a positive BAL bacterial culture or viral PCR accompanied by elevated BAL neutrophils (*N* = 2); (3) declined PET–CT, preventing categorization (*N* = 1); or (4) positive PET–CT findings of unknown significance (*N* = 2). One IGRA^+^ nonprogressor was excluded from analysis owing to presenting with a transient high PET–CT signal in the lung that quickly resolved (no. 182). Two further IGRA^+^PET–CT^+^ nonprogressors were excluded owing to lung PET signal consistent with scarring and residual inflammation from past lung disease that prevented confident PET–CT-based categorization of these individuals (nos. 048 and 233). Unless otherwise stated, data shown in figures represent the latest available BAL sample after recruitment from the indicated contact.

No flow cytometry was performed on the follow-up BAL samples from two IGRA^+^PET–CT^+^ contacts (nos. 140 and 141) because of unexpected problems with equipment. One IGRA^−^PET–CT^−^nonprogressor contact (no. 154) was not included in flow cytometry analysis of T cells, as we observed no binding of the antihuman CD4^+^ flow cytometry antibody. This same phenomenon was observed in two further contacts excluded for other reasons (nos. 097 and 098), and all three of these contacts were of African descent. In other cases, certain analyses were not performed for certain samples owing to either insufficient cell number or samples being obtained early in the study before establishment of fixed-cell scRNA-seq and T cell-focused flow cytometry methods. A full list of samples and data acquired per participant is presented in Supplementary Table 1a,b.

### PET–CT scans and analysis

^18^F-FDG PET–CT scans were performed according to local procedure guidelines at the Leicester PET–CT Centre (Alliance Medical). All participants were required to fast for 6 h before the scanning. Female participants of childbearing age were offered pregnancy testing before PET–CT. Participants received an intravenous injection of ^8^F-FDG at a dose of 3.5 MBq kg^−1^ ± 10% up to a maximum of 400 MBq. Imaging was performed from the skull vertex to the proximal third of the femur using the same PET–CT scanner (GE Discovery PET–CT 710, Alliance Medical) 1 h after radiotracer administration. Images were reconstructed using a Bayesian penalized likelihood reconstruction algorithm (Q.Clear, GE Healthcare). PET–CT scans were independently quantified by two readers. The quantification method has been described previously^51^. In brief, anonymized PET–CT DICOM images were uploaded to 3D Slicer version 5.4.0 (https://www.slicer.org). Normal physiological reference was defined as liver mean standardized uptake value (SUV) plus three standard deviations^52^. Scans were considered positive if FDG uptake exceeded the physiological reference in intrathoracic LNs and/or if focal lung parenchymal uptake was greater than background lung activity. Extracted PET metrics included maximum SUV (SUV_max_), mean SUV (SUV_mean_), metabolic volume (MV) and total lesion glycolysis (calculated by SUV_mean_ × MV). Where values differed by more than 10%, they were reviewed and re-extracted to minimize variability. Final values represent the means of scores from two independent reviewers.

### Bronchoscopy

Bronchoscopies with BAL were performed between September 2021 and October 2025 by a trained respiratory physician at University Hospitals of Leicester NHS Trust. The procedure was conducted under conscious sedation according to the British Thoracic Society guidelines for diagnostic flexible bronchoscopy^53^. Participants were required to fast for 6 h before the procedure. The flexible bronchoscope was introduced either via nasal cavity or orally through a plastic mouth guard. Sampling was directed to target lobes and lung segments identified by CXR and/or PET–CT imaging. BAL was performed by instilling and aspirating 20 ml aliquots of warmed 0.9% sodium chloride solution, up to a maximum of 250 ml, with the aim of recovering ≥60 ml of fluid (actual recovery range 37–107 ml, median 66 ml). In all participants, the first 5–10 ml of BAL fluid was sent for clinical testing including routine and mycobacterial culture and viral PCR. In participants with suspected TB, a proportion of the sample was additionally sent for cytological evaluation and tested with Xpert MTB/RIF Ultra.

### BAL processing

All sample processing, flow cytometry staining and preservation of samples for subsequent analyses was performed on the day of bronchoscopy. BAL samples were transported on gel cold packs to the Francis Crick Institute, London, UK, with a median transit time of 2 h and 50 min (range 1 h and 45 min to 4 h and 4 min) before the initiation of processing. Samples were filtered to remove mucus and debris by pipetting through 70-µm cell strainers, before pelleting by centrifugation at 500*g* for 7 min at 4 °C and transferring supernatants in aliquots to storage at −80 °C for subsequent processing. All cell pellets were briefly suspended in ammonium chloride potassium (ACK) lysing buffer (Thermo Fisher Scientific) to lyse erythrocytes, before washing with Roswell Park Memorial Institute medium (RPMI) 1640 supplemented with 10% fetal bovine serum and 5% penicillin and streptomycin (all Thermo Fisher Scientific), centrifuging at 400*g* for 5 min at 4 °C and suspending in supplemented RPMI. The numbers of cells in single-cell suspensions were counted in FastRead disposable hemocytometers (Immune Systems), with viability assessed by Trypan blue exclusion. Median viability was 82.6% (range 57.7–100%).

### RNA extraction from BAL cell pellets

Cell suspensions containing 0.5 × 10^6^ live cells were pelleted by centrifuging at 400*g* for 5 min at 4 °C and pellets suspended in TRI reagent (Sigma-Aldrich). Samples were vortexed at high speed for around 20 s before storage at −80 °C until RNA extraction. RNA extraction was performed using the DirectZol RNA Microprep kit (Zymo Technologies), as per manufacturer’s instructions, including on-column DNase I digestion.

### Bulk RNA-seq

Extracted RNA from whole BAL cell pellets was subjected to Agilent TapeStation quality control analysis and RNA integrity (RIN) values determined to be ≥7.4 and ≤9.9, except for one sample (T219_1, from a patient with active TB, no. 219), which had a RIN value of 4.1. A total of 200 ng total RNA was used for library preparation using the RNA library prep kit with Polaris ribosomal RNA depletion (Watchmaker Genomics). Approximately 2.5 × 10^7^ 100-base-pair paired-end reads were obtained per sample using an Illumina NovaSeq X sequencer.

Bulk RNA-seq data were processed and aligned to the GRCh38 genome using the default nf-core/rnaseq pipeline v3.16.1, built with Nextflow. In brief, this runs Trim Galore STAR, and Salmon for trimming, alignment and transcript count quantification, among additional quality controls. An in-depth description of the pipeline can be found at https://nf-co.re/rnaseq/3.16.1/. Normalized transcripts per million (TPM) data were then filtered for highly variable genes, defined as those with greater than twofold deviation from the median in more than 10% of samples. Hierarchical cluster analysis using Wards clustering was performed to assess major sources of variation across the dataset and led to the identification of epithelial-associated gene sets, associated with variable levels of contaminating lung epithelial material in BAL pellets (Extended Data Fig. 2a), which were removed from downstream analysis. This analysis revealed a small cluster of genes with increased expression in BAL of current tobacco smokers (Extended Data Fig. 2a). Differential gene expression (DGE) was then performed using DESeq2 v1.42.1^54^. Expression values were normalized by vst transformation and Wald tests implemented to test for differential expression between specific groups, adjusted for sex. Genes were determined to be differentially expressed with a fold change (FC) of ≥1.5 and Benjamini–Hochberg-adjusted *P* value of <0.05. DEGs were clustered on the basis of their normalized expression profile across all groups, using *k*-means clustering. Optimal *k* was determined by iterating through a *k* from 5 to 15 and manually selecting the number of clusters that best captured the observed expression pattern.

### scRNA-seq

Cell pellets containing 0.5–1 × 10^6^ live cells were fixed and permeabilized using the Chromium Next GEM Single Cell Fixed RNA Sample Preparation Kit (10X Genomics), as per manufacturer’s instructions. In brief, cell pellets were suspended in 0.04% Ultrapure BSA (Thermo Fisher Scientific) in 1× PBS (Corning), then centrifuged at 400*g* for 5 min at 4 °C, before suspending in 10X Genomics Fixation and Permeabilization buffer, supplemented with 4% molecular biology grade formaldehyde, and fixing at 4 °C for 22 h. Fixed cells were pelleted by centrifugation at 900*g* for 5 min, suspended in 10X Genomics Quench buffer and supplemented with 10X Genomics Enhancer solution and 50% molecular biology grade glycerol (Sigma-Aldrich), to a final glycerol concentration of 10%, before storing at −80 °C until subsequent batch processing. Sample were stored for between 7 and 574 days before running scRNA-seq. Fixed samples were processed in pooled scRNA-seq runs of up to ten samples using the Chromium Fixed RNA Profiling Kits for Multiplexed Samples, splitting each sample over one to three barcoded probe sets, to maximize cells recovered per sample where input cell number was sufficient for splitting over multiple barcoded probe sets.

### scRNA-seq analysis

Cell Ranger v7.0.1 raw matrices were input into CellBender v0.3.2 to model ambient RNA and remove contaminating transcripts^55^. The expected number of cells per sample was set to recover cell numbers comparable to original input. Filtered matrices from CellBender were then processed in Python v3.11.13 using scanpy v1.11.4^56^. Cells were filtered using a permissive lower cutoff of 100 total UMI counts or 100 unique genes after ambient RNA removal, and a mitochondrial transcript fraction greater than 5%, on the basis of iterative quality control checks to retain populations with low RNA content such as neutrophils. Scrublet^57^ was then used to identify likely doublet cells. Cells marked as doublets were not excluded from the initial analysis but were instead used to identify doublet-enriched clusters downstream. Log normalization and principal component (PC) analysis were performed using scanpy functions sc.pp.normalize_total, sc.pp.log1p and sc.tl.pca.

An integrated manifold was generated using the scanpy Harmony implementation^58^ with key set to ‘sample’, using the top 2,000 variable features and first 50 PCs. The Harmony-corrected PCs were then used to generate a nearest-neighbor graph and uniform manifold approximation and projection (UMAP) embedding.

On the basis of the nearest-neighbor graph, single cells were annotated according to similarity to reference populations in published human lung and immune cell scRNA-seq datasets^59,60^ using CellTypist^59^. Iterative Leiden clustering to delineate cell types and recover cell populations reflected in the CellTypist annotation was performed, during which low-quality clusters were removed. A single doublet cluster (10,087 cells) exhibiting higher Scrublet score as well as co-expression of macrophage and T-cell-lineage genes was removed. As epithelial cells reflected variable, but low level, contaminants in BAL samples, 11,282 cells within clusters annotated as epithelial by CellTypist were also excluded before reclustering leukocytes only. A clustering resolution of 1.4 was applied, before further manual adjustments to either resolve specific cell types annotated by CellTypist or to resolve observed heterogeneity within neutrophils (0.5 for T and NK cells, 0.3 for neutrophils, 0.15 for conventional DCs and 0.15 to resolve plasmacytoid DCs, mast cells and B cells). The resultant 28 clusters were annotated on the basis of CellTypist analysis against three reference datasets^59–61^ with additional refinements based on marker gene expression (Supplementary Table 4) that was essential to identify neutrophils, which were not well represented in these reference datasets.

### Differential abundance

Differential abundance of major cell types across conditions was assessed using the cluster-agnostic statistical tool Milo^62^ via the Python perturbation framework Pertpy v1.0.2^63^, using parameters *k* = 150, *P* = 0.1 and *d* = 50. Milo defines a set of representative neighborhoods on the *k*-nearest neighbor graph of single cells to model neighborhood abundance, while accounting for the compositional nature of cell count data. Differentially abundant neighborhoods were visualized on the full UMAP or mapped back to their majority cell types (those neighborhoods with >60% cell type labeled) and visualized according to log_2_ FC between comparisons.

### Single-cell DGE

Pseudobulk DGE analysis was carried out using the decoupler package v2.1.1^64^ to generate the pseudobulks and the Python implementation of DESeq2, PyDESeq2 v0.5.2, for the differential expression^54,65^. In brief, gene expression was aggregated per individual and per cell type. Genes were filtered on the basis of minimum expression of *n* counts in at least 15% of *X* samples, where *X* was the smallest group in the comparison and *n* was defined for each cell type individually. DGE analysis was then computed using Wald tests implemented with PyDESeq2, with models adjusted for sex. Genes were determined to be differentially expressed with an FC of ≥1.5 and Benjamini–Hochberg-adjusted *P* value of <0.05. Pathway enrichment analysis of DEGs was performed using overrepresentation analysis with GSEA.py against the MSigDB Hallmark 2020 pathway database, implementing a hypergeometric overrepresentation test with Benjamini–Hochberg correction for multiple testing.

### Inference of cell-to-cell communication from scRNA-seq data

Cell-to-cell interactions were inferred using the R package CellChat v1.1.3, with default parameters. The ‘population.size’ parameter was set to TRUE when computing the inferred interaction between cell subsets. Relative predicted signaling contribution of different ligand–receptor pairs and cell types was quantified as indicated in the relevant figures.

### Gene signature evaluation in scRNA-seq data

Enrichment of gene signatures was scored in cells using the scanpy function sc.tl.score_genes. External gene signatures for evaluation in our scRNA-seq data were obtained from the top 50 genes of a protective T1–T17 T cell signature from NHP granuloma^15^ and from determining common type I IFN response genes from in vitro type I IFN-stimulated human neutrophils^66^ and human and mouse macrophages^67^. A random forest model was used to rank genes within the published gene signatures according to their contribution to calculating the enrichment score. A signature of inflammatory macrophages in neutrophil-high patients with TB and progressors was obtained by *k*-means clustering of DEGs and reduced to the top 50 DEGs by adjusted *P* value across relevant clusters for use in enrichment analysis.

### Analysis of external bulk and scRNA-seq data

Published scRNA-seq data from cynomolgus macaque TB granulomas^13^ were obtained from the Broad Single Cell Portal (SRA: PRJNA900256) and the processed dataset imported and analyzed in Python. Transcriptional signatures derived from our human BAL data were also assessed in our previously published scRNA-seq dataset from lung leukocytes of TB-resistant and -susceptible mouse strains during *M. tuberculosis* infection^21^ (GSE298787) and in bulk RNA-seq data from whole mouse lungs from TB-susceptible mice during *M. tuberculosis* infection with and without IFNAR blockade^21^ (GSE298786).

### Flow cytometry

Approximately 10^6^ live cells per staining panel were washed in PBS, before staining for 30 min at room temperature with amine-reactive Fixable Blue Viability Dye (Thermo Fisher Scientific; diluted 1 in 500 in PBS). Samples were incubated with 25 mg ml^−1^ Human Fc Block (BD Biosciences) diluted in staining buffer (PBS + 2% fetal bovine serum + 2 mM EDTA) for 5 min at room temperature, before the addition of cocktails of fluorochrome-conjugated monoclonal antibodies; either for pan-leukocyte analysis or T cell-focused analysis, supplemented with True-Stain Monocyte Blocker (BioLegend), diluted 1:50, to prevent nonspecific binding of tandem fluorochromes to monocytes and macrophages. The pan-leukocyte panel comprised the antibodies (RRID, then dilution, in parentheses): HLA-DR-BV785 (AB_2563461, 1:100), CD206-APC/Cy7 (AB_2144930, 1:100), CD45-PERCP/Cy5.5 (AB_893338, 1:200), CD16-AF700 (AB_2278418, 1:200), CD11c-PE/Cy7 (AB_389351, 1:200), CD14-PE (AB_314188, 1:200), CD15-FITC (AB_314196, 1:200), CD3-BV510 (AB_2561943, 1:200), CD56-APC (AB_2563913, 1:200), CD19-BV650 (AB_2562097, 1:400) and CD4-PE/Dazzle595 (AB_2565847, 1:400) from BioLegend and CD8a-BUV737 (AB_2870085, 1:400) and Siglec 8-BV711 (AB_2872332, 1:200) from BD Biosciences. The T cell-focused phenotyping panel comprised the antibodies: CCR7-BV711 (AB_2563865, 1:50), CCR6-PE/Cy7 (AB_10916518, 1:100), CXCR3-APC (AB_10983064, 1:100), PD-1-BV650 (AB_2566362, 1:100), HLA-DR-FITC (AB_314682, 1:100), CD69-APC/Cy7 (AB_314849, 1:200), CD45-PERCP/Cy5.5 (AB_893338, 1:200), CD45RA-PE (AB_314412, 1:200), CD3-BV510 (AB_2561943, 1:200), CD56-BV785 (AB_2566059, 1:200), CD103-PE/Dazzle594 (AB_2716189, 1:200), CD4-AF700 (AB_571943, 1:50) or CD4-PE/Cy7 (AB_571959, 1:400) from BioLegend and CD8a-BUV737 (AB_2870085, 1:400) from BD Biosciences. Clone and lot number information for flow cytometry antibodies are presented in Supplementary Table 8.

Flow cytometry data were acquired on an X20 analyzer using FACSDiva v9.2 software (BD Biosciences). Autofluorescence was measured in the 450-nm 450/50 channel and used as an analysis parameter. Data were analyzed using FlowJo v10 software (BD Biosciences). Populations were defined by hierarchical gating as shown in Extended Data Figs. 3a and 7a and expressed as either percentage of total leukocytes (defined as live, CD45^+^, single cells), percentage of a parent population or as absolute counts (calculated as no. of cell population / no. of live single cells × no. of live cells per milliliter BAL), as counted manually on a hemocytometer.

### Luminex protein assays

BAL supernatants were maintained at −80 °C for between 21 and 119 weeks before thawing and sterilizing by centrifugation at 10,000*g* through Ultrafree 0.22-um centrifugal filters (Sigma-Aldrich). Three custom Luminex Discovery Assays were designed and purchased from Bio-Techne and were performed as per manufacturer’s instructions. Samples were run at a minimum of 1:1 dilution with assay buffer. Data were acquired on a Bio-Plex 200 system (Bio-Rad) and concentration values interpolated from standard curves using Bio-Plex Manager software (Bio-Rad). Inferred values below the bottom standard concentration were ignored and interpreted as below detection limit. Values below the effective limit of detection (bottom standard concentration multiplied by 2, the lowest sample dilution factor of BAL supernatants tested) or identical values close to the effective limit of detection obtained for several samples were interpreted as likely noise and so recorded as below detection limit. Assays consisted of (1) CCL1, CCL2, CCL3*, CCL4, CCL5, CCL13, CCL14, CCL15*, CCL19, CCL20, CCL23*, CCL24, CCL25*, CCL26*, CCL27*, CCL28*, CX_3_CL1*, CXCL2*, CXCL6, CXCL12*; (2) C-reactive protein, CXCL9, G-CSF, GITR*, GM-CSF, Granzyme B, IFN-α, IFN-β*, IFN-γ, IL-1α, IL-1β, IL-RA, IL-2*, IL-3*, IL-4*, IL-5*, IL-10,* IL-11*, IL-12/23p40*, IL-17A*, IL-17C, IL-18, IL-19, IL-21*, IL-28A*, IL-28B*, MMP-1, MMP-12 and TNF; (3) CXCL10, CCL18, CXCL16, IL-8, IL-6, MMP-2, MMP-9, MMP-8, CXCL11, CCL17, CCL22. Asterisks in the above lists indicate analytes that were not present above the limit of detection in at least eight samples and so were not included in the presented analysis. C-reactive protein data were not included in the presented analysis, as values were over the limit of detection in several samples. Luminex data were visualized on a row-scaled heat map generated using the pheatmap v1.0.13 R package using clustering method ‘ward.D2’.

### Blinding

Sample collection and initial processing was performed by operators aware of the clinical status of participants at the point of sampling, and so blinding was not possible for this study. RNA extraction for bulk RNA-seq was performed in batches in the order of sample collection while blinded to final patient group. scRNA-seq runs of pooled samples were performed in parallel to sample collection, and so pool composition was determined on the basis of similar sampling date and on similar cellular composition of BAL as determined by flow cytometry, following our preliminary observations that this improved the balance of sequencing coverage across samples within pools. The operator preparing and running scRNA-seq pools was blind to the study group. Bulk and scRNA-seq library preparation was performed through automated core facility pipelines at the Francis Crick Institute by operators blinded to the study groups.

### Statistical analysis

Unless otherwise specified, statistical analysis was performed using Prism v10.6.0 software (GraphPad) using appropriate statistical tests for the number of groups analyzed and the distribution of data, which was determined using Shapiro–Wilk normality tests in Prism. Two-tailed tests were used. Descriptive statistics, statistical tests and results are specified in the relevant figures and accompanying legends.

### Reporting summary

Further information on research design is available in the Nature Portfolio Reporting Summary linked to this article.

## Online content

Any methods, additional references, Nature Portfolio reporting summaries, source data, extended data, supplementary information, acknowledgements, peer review information; details of author contributions and competing interests; and statements of data and code availability are available at 10.1038/s41590-026-02544-0.

## Supplementary information

Supplementary Information Supplementary Fig. 1. PET–CT scan images from all contacts who received bronchoscopy. **a**, All household contacts progressing to TB. **b**, All IGRA^+^ nonprogressor contacts. **c**, All IGRA^−^ nonprogressor contacts.

Reporting Summary

Supplementary Tables 1–8. Supplementary Table 1. Details of study participants and samples obtained. **a**, All TB household contacts, including progressors. **b**, All patients with TB. **c**, Full details on progressing contacts. Supplementary Table 2. Lists of genes in the hierarchically clustered heat map of whole BAL bulk RNA-seq data in Extended Data Fig. 2a. Supplementary Table 3. Lists of genes in the *k*-means clusters of whole BAL bulk RNA-seq differential expression analysis in Extended Data Fig. 2b. Supplementary Table 4. Details of scRNA-seq cluster annotations. Supplementary Table 5. Numbers and proportions of scRNA-seq clusters per sample. Supplementary Table 6. Lists of DEGs in scRNA-seq BAL leukocyte populations from neutrophil-high compared with neutrophil-low patients with TB and progressors (accompanies Figs. 4 and 6). Supplementary Table 7. Lists of DEGs in scRNA-seq BAL leukocyte populations from comparisons between subgroups of nonprogressor and progressor contacts (accompanies Fig. 7). Supplementary Table 8. Details of flow cytometry antibodies used in the study.

## Data Availability

Bulk RNA-seq data generated in this study have been deposited in the Gene Expression Omnibus (GEO) with accession no. GSE328391; scRNA-seq data generated in this study have been deposited in the GEO with accession no. GSE326212. Requests for other data types should be directed to the corresponding author.

## Code availability

All code to reproduce the analysis is available via GitHub at https://github.com/ogarralab/branchett_human_bal_tb_2026 and via Zenodo at 10.5281/zenodo.19609843 (ref. ^68^).
