# Supplementary material for: Airway immune signatures of protection and disease progression in recent human tuberculosis household contacts
Source: Nat Immunol. 2026 Jun 24;27(8):1577–89. doi: 10.1038/s41590-026-02544-0 (PMC13414559; doi:10.1038/s41590-026-02544-0)
Supplement: Supplementary file 1 — Supplementary Fig. 1. PET–CT scan images from all contacts who received bronchoscopy. a, All household contacts progressing to TB. b, All IGRA+ nonprogressor contacts. c, All IGRA− nonprogressor contacts. [file 41590_2026_2544_MOESM1_ESM.pdf]

nature immunology

Supplementary information

https://doi.org/10.1038/s41590-026-02544-0

Airway immune signatures of protection and disease progression in recent human tuberculosis household contacts

In the format provided by the authors and unedited

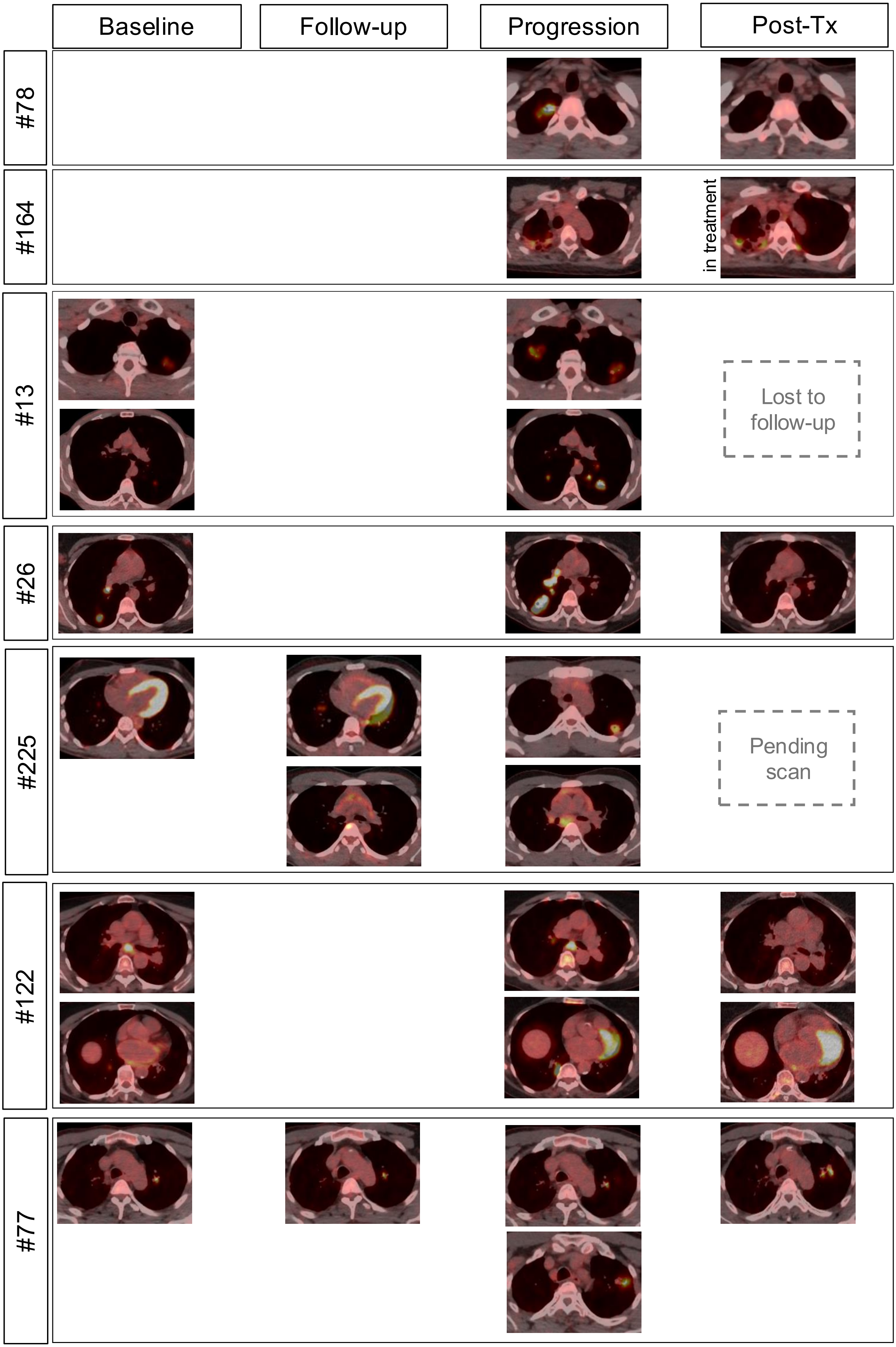

Supplementary Information Fig. 1a. Thoracic PET-CT scans of progressing contacts

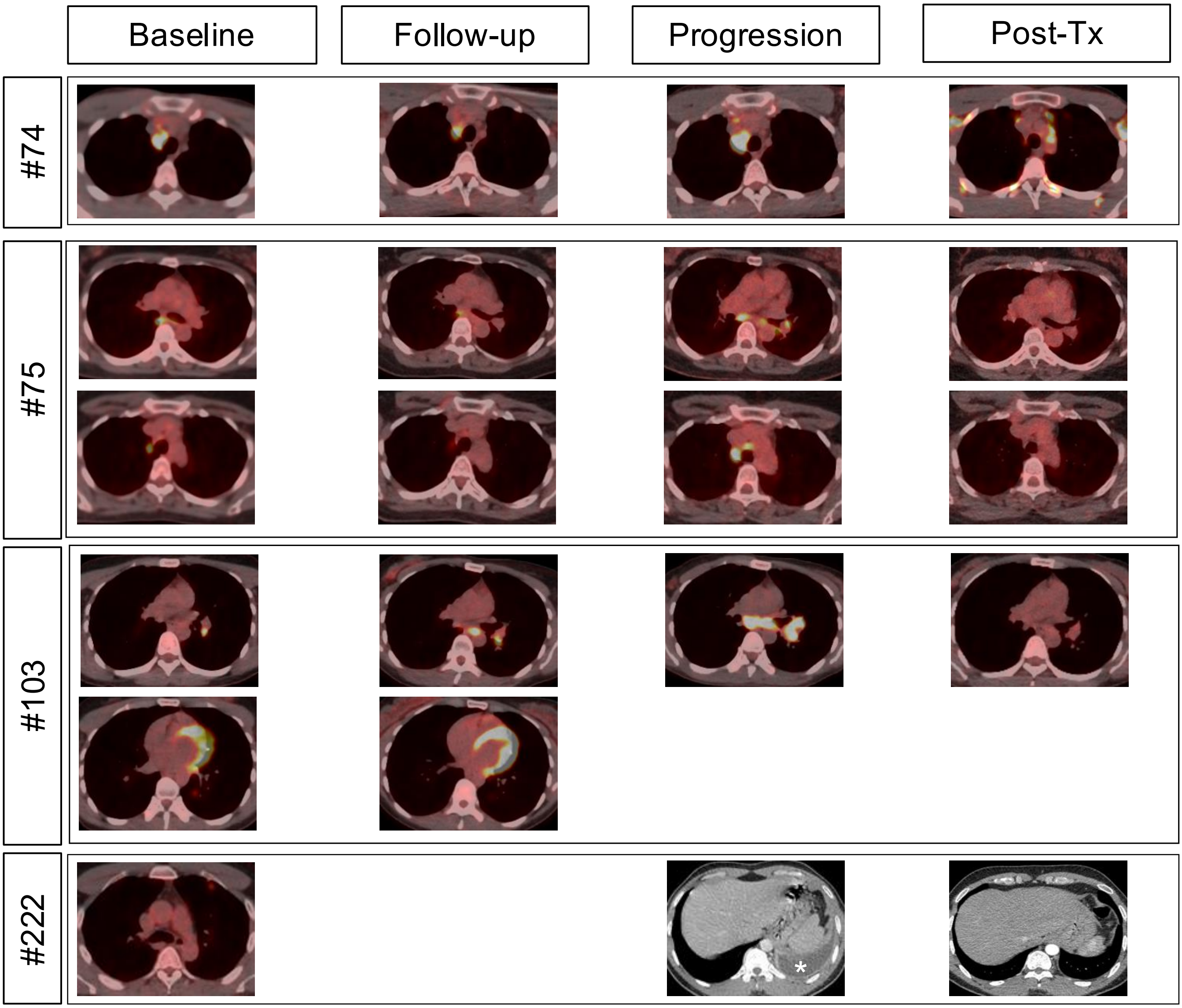

Supplementary Information Fig. 1a (continued). Thoracic PET-CT or CT only images from progressing contacts. *, indicates pleural effusion on CT of one progressor that resolved with treatment.

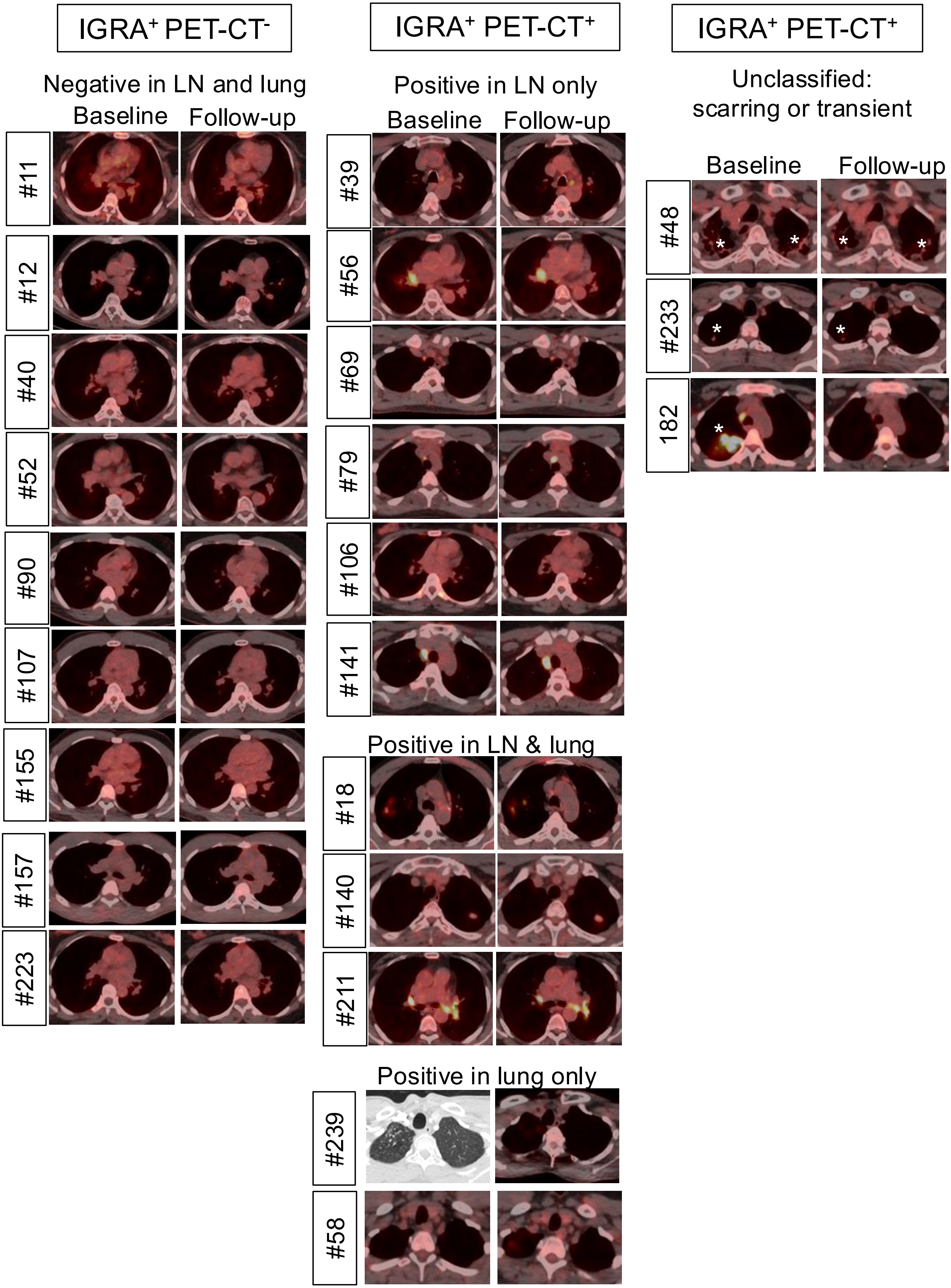

Supplementary Information Fig. 1b. Thoracic PET-CT or CT only images in IGRA+ non-progressors. *, indicates scarring or transient positive PET-CT signal

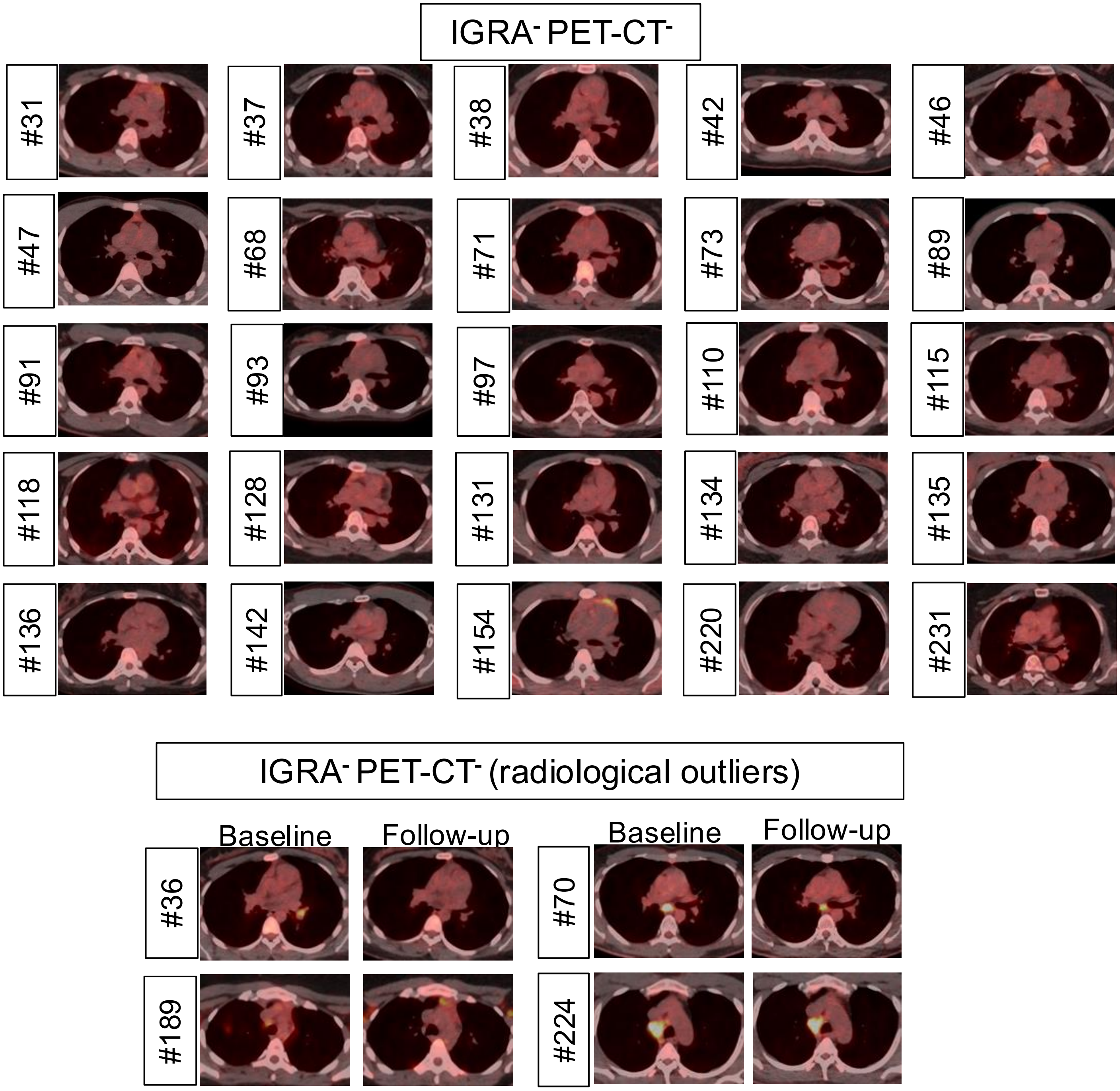

Supplementary Information Fig. 1c Thoracic PET-CT images in IGRA^{-} non-progressors.
