# Supplementary material for: Airway immune signatures of protection and disease progression in recent human tuberculosis household contacts
Source: Nat Immunol. 2026 Jun 24;27(8):1577–89. doi: 10.1038/s41590-026-02544-0 (PMC13414559; doi:10.1038/s41590-026-02544-0)
Supplement: Supplementary file 2 — Reporting Summary [file 41590_2026_2544_MOESM2_ESM.pdf]

nature portfolio

Corresponding author(s): Dr Anne O'Garra

Last updated by author(s): Apr 30, 2026

Reporting Summary

Nature Portfolio wishes to improve the reproducibility of the work that we publish. This form provides structure for consistency and transparency in reporting. For further information on Nature Portfolio policies, see our Editorial Policies and the Editorial Policy Checklist.

Statistics

For all statistical analyses, confirm that the following items are present in the figure legend, table legend, main text, or Methods section.

n/a	Confirmed	
☐	☒	The exact sample size (*n*) for each experimental group/condition, given as a discrete number and unit of measurement
☐	☒	A statement on whether measurements were taken from distinct samples or whether the same sample was measured repeatedly
☐	☒	The statistical test(s) used AND whether they are one- or two-sided<br>*Only common tests should be described solely by name; describe more complex techniques in the Methods section.*
☐	☒	A description of all covariates tested
☐	☒	A description of any assumptions or corrections, such as tests of normality and adjustment for multiple comparisons
☐	☒	A full description of the statistical parameters including central tendency (e.g. means) or other basic estimates (e.g. regression coefficient) AND variation (e.g. standard deviation) or associated estimates of uncertainty (e.g. confidence intervals)
☐	☒	For null hypothesis testing, the test statistic (e.g. *F*, *t*, *r*) with confidence intervals, effect sizes, degrees of freedom and *P* value noted<br>*Give P values as exact values whenever suitable.*
☒	☐	For Bayesian analysis, information on the choice of priors and Markov chain Monte Carlo settings
☒	☐	For hierarchical and complex designs, identification of the appropriate level for tests and full reporting of outcomes
☐	☒	Estimates of effect sizes (e.g. Cohen's *d*, Pearson's *r*), indicating how they were calculated

Our web collection on statistics for biologists contains articles on many of the points above.

Software and code

Policy information about availability of computer code

Data collection	Flow cytometry data were acquired using BD FACSDiva software v9.2; Luminex protein assay data were acquired using Bio-Plex Manager v6.0 software
Data analysis	PET-CT data were analysed using 3D Slicer v5.4.0; flow cytometry data were analysed using FlowJo v10 software; interpolation of Luminex protein assay data was performed using Bio-Plex Manager v6.0 software, with additional plotting in GraphPad Prism v10.6.0 and clustering using pheatmap v1.0.13 in R; bulk RNA-seq data processing and alignment was performing using the Nextflow v3.16.1 RNA-seq pipeline; differential expression analysis of bulk RNA-seq data was performed using DESeq2 v1.42.1; cell calling and ambient RNA removal from scRNA-seq data were performed using CellBender v0.3.2; further processing and clustering of scRNA-seq data were performed in scanpy v1.11.4 in Python; doublet identification was performed using Scrublet; CellTypist was used to annotate cell populations in scRNA-seq data based on similarity to reference populations; differential abundance analysis of scRNA-seq data was performed using Milo via Pertpy v1.0.2; pseudobulk generation from scRNA-seq data was performed using decoupler v2.1.1; differential expression analysis on pseudobulks was performed using PyDESeq2 v0.5.2 in Python; cell-cell interaction prediction analysis was performed using CellChat v1.1.2 in R; GraphPad Prism v10.6.0 was used for all other statistical analysis.

For manuscripts utilizing custom algorithms or software that are central to the research but not yet described in published literature, software must be made available to editors and reviewers. We strongly encourage code deposition in a community repository (e.g. GitHub). See the Nature Portfolio guidelines for submitting code & software for further information.

Data

Policy information about availability of data

All manuscripts must include a data availability statement. This statement should provide the following information, where applicable:

- Accession codes, unique identifiers, or web links for publicly available datasets
- A description of any restrictions on data availability
- For clinical datasets or third party data, please ensure that the statement adheres to our policy

Bulk RNA-seq data generated in this study have been deposited in the GEO with accession GSE328391; scRNA-seq data generated in this study have been deposited in the GEO with accession GSE326212. Requests for other data types should be directed to the corresponding author.

Research involving human participants, their data, or biological material

Policy information about studies with human participants or human data. See also policy information about sex, gender (identity/presentation), and sexual orientation and race, ethnicity and racism.

Reporting on sex and gender	Participants of both male and female sex were recruited to our study and the breakdown of male and female participants per group is included in Supplementary Table 1. Differential gene expression analysis included an adjustment for the effects of sex.
Reporting on race, ethnicity, or other socially relevant groupings	Broad ethnicity data for participants as recorded in the clinic is included to allow assessment of any impact of ethnicity on the immune responses and signatures measured in our study. We did not observe substantial effects of ethnicity in our analyses and so did not adjust for ethnicity in our differential gene expression analysis.
Population characteristics	This is detailed in the Methods and in Supplementary Table 1. Participants ranged from 17 to 68 years of age at the point of recruitment. Participants were predominantly South Asian (67%), but also included participants identified as Other Asian (9.4%), White British (6.3%), Other White (9.4%), African (6.3%), Caribbean (1%) and Arab (1%).
Recruitment	Participant recruitment was based on predefined inclusion and exclusion criteria as described in full in our Methods. Participants were recruited through the University Hospitals of Leicester NHS Trust and so are representative of our study site, without self-selection bias.
Ethics oversight	The study was approved by the Research and Ethics Committee (REC) for East Midlands - Nottingham 1, Nottingham, UK (REC

Note that full information on the approval of the study protocol must also be provided in the manuscript.

Field-specific reporting

Please select the one below that is the best fit for your research. If you are not sure, read the appropriate sections before making your selection.

☒ Life sciences ☐ Behavioural & social sciences ☐ Ecological, evolutionary & environmental sciences

For a reference copy of the document with all sections, see nature.com/documents/nr-reporting-summary-flat.pdf

Life sciences study design

All studies must disclose on these points even when the disclosure is negative.

Sample size	A target of 30 active TB patients was selected based on our past experience working with blood transcriptional signatures of TB patients.<br><br>All IGRA-positive TB contacts who could be recruited were recruited to the study, with 10 progressors identified as a target in our power calculations prior to starting the study. Based on our past studies of blood transcriptional signatures in TB contacts, a sample size providing 10 progressors was expected to give at least 80% power to detect an airway transcriptional signature of progression in 5 of 10 progressors.
Data exclusions	Data exclusions and data types obtained per participant are described in full in the Methods and Supplementary Table 1. Exclusions were principally of clinical outliers with evidence of potentially confounding infections or other conditions that became apparent after sampling. Current tobacco smokers were excluded from analysis of non-progressor contact groups, due to potential confounding effects of smoking on the subtle immune signatures expected in non-progressors.
Replication	This is a unique study conducted over 5 years using PET-CT and bronchoalveolar lavage to define immunological changes in the airways of TB contacts who either progress to TB or remain healthy, all in a low TB burden setting. No other comparable cohort is available in which to replicate our study, which due to the participant number and robustness of the data showed reproducible data across participants.
Randomization	Randomization was not applicable to this prospective observational cohort study as no experimental interventions were applied and study groups were defined by the participants' clinical status.
Blinding	Sample collection and initial processing was performed by operators aware of the clinical status of participants at the point of sampling and so blinding was not possible for this study. RNA extraction for bulk RNA-seq was performed in batches in the order of sample collection while

blind to final patient group. Single cell RNA-seq runs of pooled samples were performed in parallel to sample collection and so pool composition was determined based on similar sampling date and on similar cellular composition of BAL as determined by flow cytometry, following our preliminary observations that this improved the balance of sequencing coverage across samples within pools. The operator preparing and running scRNA-seq pools was blind to the study group. Bulk and scRNA-seq library preparation was performed through automated core facility pipelines at the Francis Crick Institute by operators blind to the study groups.

Reporting for specific materials, systems and methods

We require information from authors about some types of materials, experimental systems and methods used in many studies. Here, indicate whether each material, system or method listed is relevant to your study. If you are not sure if a list item applies to your research, read the appropriate section before selecting a response.

Materials & experimental systems

n/a	Involved in the study	
☐	☒	Antibodies
☐	☐	Eukaryotic cell lines
☐	☐	Palaeontology and archaeology
☐	☐	Animals and other organisms
☐	☒	Clinical data
☐	☐	Dual use research of concern
☐	☐	Plants

Methods

n/a	Involved in the study	
☐	☐	ChIP-seq
☐	☒	Flow cytometry
☐	☐	MRI-based neuroimaging

Antibodies

Antibodies used

The pan-leukocyte flow cytometry panel comprised the antibodies (RRID, then dilution, then lots, in parentheses): HLA-DR-BV785 (AB_2563461, 1:100, 1222393; 2075800; 2313207), CD206-APC/Cy7 (AB_2144930, 1:100, B301406; B351612), CD45-PERCP/Cy5.5 (AB_893338, 1:200, B275271; B350438; B384536), CD16-AF700 (AB_2278418, 1:200, B333714; B335559), CD11c-PE/Cy7 (AB_389351, 1:200, B312100; B308581), CD14-PE (AB_314188, 1:200, B287099), CD15-FITC (AB_314196, 1:200, B320428), CD3-BV510 (AB_2561943, 1:200, B333191; B305282; B319035; B372273), CD56-APC (AB_2563913, 1:200, B303959; B369154), CD19-BV650 (AB_2562097, 1:400, B300888; B337275), and CD4-PE/Dazzle594 (AB_2565847, 1:400, B304434; B316533) from BioLegend and CD8a-BUV737 (AB_2870085, 1:400, 311372; 1125204; 1197655; 2221885) and Siglec 8-BV711 (AB_2872332, 1:200, 1222393; 2075800; 2313207) from BD Biosciences. The T cell-focused phenotyping panel comprised the antibodies: CCR7-BV711 (AB_2563865, 1:50, B303460; B346154; B367229), CCR6-PE/Cy7 (AB_10916518, 1:100, B320791; B346873), CXCR3-APC (AB_10983064, 1:100, B314796; B330515), PD-1-BV650 (AB_2566362, 1:100, B295100; B341605), HLA-DR-FITC (AB_314682, 1:100, B275368), CD69-APC/Cy7 (AB_314849, 1:200, B284261; B330645; B360871), CD45-PERCP/Cy5.5 (AB_893338, 1:200, B275271; B350438; B384536), CD45RA-PE (AB_314412, 1:200, B286714; B324778; B352674), CD3-BV510 (AB_2561943, 1:200, B333191; B305282; B319035; B372273), CD56-BV785 (AB_2566059, 1:200, B303959; B369154), CD103-PE/Dazzle594 (AB_2716189, 1:200, B278649; B321310; B351616), CD4-AF700 (AB_571943, 1:50, B247388; B346479) or CD4-PE/Cy7 (AB_571959, 1:400, B278099) from BioLegend and CD8a-BUV737 (AB_2870085, 1:400, 311372; 1125204; 1197655; 2221885) from BD Biosciences. Full antibody information is provided in Supplementary Table 8.

Validation

Verification for all flow cytometry antibodies was based on the manufacturer's validation as detailed on their website and previous literature reporting use of these antibodies, examples of which are provided on the manufacturer websites. Many of our flow cytometry antibodies were selected based on extensive past use for human bronchoalveolar lavage staining by our collaborators (e.g. Byrne et al., J. Exp. Med, 2020; doi: 10.1084/jem.20191236)

Verification of the Luminex antibodies was based on internal verification from the supplier and the kit was used exactly as per the manufacturer's instructions.

Eukaryotic cell lines

Policy information about cell lines and Sex and Gender in Research

Cell line source(s)

State the source of each cell line used and the sex of all primary cell lines and cells derived from human participants or vertebrate models.

Authentication

Describe the authentication procedures for each cell line used OR declare that none of the cell lines used were authenticated.

Mycoplasma contamination

Confirm that all cell lines tested negative for mycoplasma contamination OR describe the results of the testing for mycoplasma contamination OR declare that the cell lines were not tested for mycoplasma contamination.

Commonly misidentified lines
(See ICLAC register)

Name any commonly misidentified cell lines used in the study and provide a rationale for their use.

Palaeontology and Archaeology

Specimen provenance	*Provide provenance information for specimens and describe permits that were obtained for the work (including the name of the issuing authority, the date of issue, and any identifying information). Permits should encompass collection and, where applicable, export.*
Specimen deposition	*Indicate where the specimens have been deposited to permit free access by other researchers.*
Dating methods	*If new dates are provided, describe how they were obtained (e.g. collection, storage, sample pretreatment and measurement), where they were obtained (i.e. lab name), the calibration program and the protocol for quality assurance OR state that no new dates are provided.*

☐ Tick this box to confirm that the raw and calibrated dates are available in the paper or in Supplementary Information.

Ethics oversight	*Identify the organization(s) that approved or provided guidance on the study protocol, OR state that no ethical approval or guidance was required and explain why not.*

Note that full information on the approval of the study protocol must also be provided in the manuscript.

Animals and other research organisms

Policy information about studies involving animals; ARRIVE guidelines recommended for reporting animal research, and Sex and Gender in Research

Laboratory animals	*For laboratory animals, report species, strain and age OR state that the study did not involve laboratory animals.*
Wild animals	*Provide details on animals observed in or captured in the field; report species and age where possible. Describe how animals were caught and transported and what happened to captive animals after the study (if killed, explain why and describe method; if released, say where and when) OR state that the study did not involve wild animals.*
Reporting on sex	*Indicate if findings apply to only one sex; describe whether sex was considered in study design, methods used for assigning sex. Provide data disaggregated for sex where this information has been collected in the source data as appropriate; provide overall numbers in this Reporting Summary. Please state if this information has not been collected. Report sex-based analyses where performed, justify reasons for lack of sex-based analysis.*
Field-collected samples	*For laboratory work with field-collected samples, describe all relevant parameters such as housing, maintenance, temperature, photoperiod and end-of-experiment protocol OR state that the study did not involve samples collected from the field.*
Ethics oversight	*Identify the organization(s) that approved or provided guidance on the study protocol, OR state that no ethical approval or guidance was required and explain why not.*

Note that full information on the approval of the study protocol must also be provided in the manuscript.

Clinical data

Policy information about clinical studies

All manuscripts should comply with the ICMJE guidelines for publication of clinical research and a completed CONSORT checklist must be included with all submissions.

Clinical trial registration	This was not a clinical trial, but was a prospective observational cohort study in human participants, which included collection of clinical data. This study therefore required registration on the UK ISRCTN (ISRCTN1798557) but is not a clinical trial.
Study protocol	Not applicable as not a clinical trial
Data collection	This was a 24-month prospective observational cohort study of pulmonary TB patients and recent household TB contacts, recruited between September 2021 and April 2024 at University Hospitals of Leicester NHS Trust, UK
Outcomes	Not applicable as not a clinical trial

Dual use research of concern

Policy information about dual use research of concern

Hazards

Could the accidental, deliberate or reckless misuse of agents or technologies generated in the work, or the application of information presented in the manuscript, pose a threat to:

No	Yes	
☐	☐	Public health
☐	☐	National security
☐	☐	Crops and/or livestock
☐	☐	Ecosystems
☐	☐	Any other significant area

Experiments of concern

Does the work involve any of these experiments of concern:

No	Yes	
☐	☐	Demonstrate how to render a vaccine ineffective
☐	☐	Confer resistance to therapeutically useful antibiotics or antiviral agents
☐	☐	Enhance the virulence of a pathogen or render a nonpathogen virulent
☐	☐	Increase transmissibility of a pathogen
☐	☐	Alter the host range of a pathogen
☐	☐	Enable evasion of diagnostic/detection modalities
☐	☐	Enable the weaponization of a biological agent or toxin
☐	☐	Any other potentially harmful combination of experiments and agents

Plants

Seed stocks	*Report on the source of all seed stocks or other plant material used. If applicable, state the seed stock centre and catalogue number. If plant specimens were collected from the field, describe the collection location, date and sampling procedures.*
Novel plant genotypes	*Describe the methods by which all novel plant genotypes were produced. This includes those generated by transgenic approaches, gene editing, chemical/radiation-based mutagenesis and hybridization. For transgenic lines, describe the transformation method, the number of independent lines analyzed and the generation upon which experiments were performed. For gene-edited lines, describe the editor used, the endogenous sequence targeted for editing, the targeting guide RNA sequence (if applicable) and how the editor was applied.*
Authentication	*Describe any authentication procedures for each seed stock used or novel genotype generated. Describe any experiments used to assess the effect of a mutation and, where applicable, how potential secondary effects (e.g. second site T-DNA insertions, mosiacism, off-target gene editing) were examined.*

ChIP-seq

Data deposition

☐ Confirm that both raw and final processed data have been deposited in a public database such as GEO.

☐ Confirm that you have deposited or provided access to graph files (e.g. BED files) for the called peaks.

Data access links<br>*May remain private before publication.*	*For "Initial submission" or "Revised version" documents, provide reviewer access links. For your "Final submission" document, provide a link to the deposited data.*
Files in database submission	*Provide a list of all files available in the database submission.*
Genome browser session<br>(e.g. UCSC)	*Provide a link to an anonymized genome browser session for "Initial submission" and "Revised version" documents only, to enable peer review. Write "no longer applicable" for "Final submission" documents.*

Methodology

Replicates	*Describe the experimental replicates, specifying number, type and replicate agreement.*
Sequencing depth	*Describe the sequencing depth for each experiment, providing the total number of reads, uniquely mapped reads, length of reads and whether they were paired- or single-end.*
Antibodies	*Describe the antibodies used for the ChIP-seq experiments; as applicable, provide supplier name, catalog number, clone name, and lot number.*
Peak calling parameters	*Specify the command line program and parameters used for read mapping and peak calling, including the ChIP, control and index files used.*

Data quality | *Describe the methods used to ensure data quality in full detail, including how many peaks are at FDR 5% and above 5-fold enrichment.*

Software | *Describe the software used to collect and analyze the ChIP-seq data. For custom code that has been deposited into a community repository, provide accession details.*

Flow Cytometry

Plots

Confirm that:

☒ The axis labels state the marker and fluorochrome used (e.g. CD4-FITC).

☒ The axis scales are clearly visible. Include numbers along axes only for bottom left plot of group (a 'group' is an analysis of identical markers).

☒ All plots are contour plots with outliers or pseudocolor plots.

☒ A numerical value for number of cells or percentage (with statistics) is provided.

Methodology

Sample preparation	Approximately 106 live cells per staining panel were washed in PBS, before staining for 30 mins at room temperature with amine-reactive Fixable Blue Viability Dye (Thermo Fisher Scientific; diluted 1 in 500 in PBS). Samples were incubated with 25 mg/ml Human Fc Block (BD Biosciences) diluted in staining buffer (PBS + 2% fetal bovine serum + 2mM EDTA) for 5 minutes at room temperature, before addition of cocktails of fluorochrome-conjugated monoclonal antibodies; either for pan-leukocyte analysis or T cell-focused analysis, supplemented with True-Stain Monocyte Blocker (BioLegend), diluted 1:50, to prevent non-specific binding of tandem fluorochromes to monocytes and macrophages.
Instrument	BD X20
Software	Acquisition: BD FACSDiva v9.2<br>Analysis: FlowJo v10
Cell population abundance	Not applicable (samples were not sorted)
Gating strategy	Initial gating was on singlet (based on FSC-A vs FSC-H), live (dead cell dye-negative), CD45-positive cells, before gating leukocyte populations as defined in Extended Data Figures 3a and 6a.

☒ Tick this box to confirm that a figure exemplifying the gating strategy is provided in the Supplementary Information.

Magnetic resonance imaging

Experimental design

Design type	*Indicate task or resting state; event-related or block design.*
Design specifications	*Specify the number of blocks, trials or experimental units per session and/or subject, and specify the length of each trial or block (if trials are blocked) and interval between trials.*
Behavioral performance measures	*State number and/or type of variables recorded (e.g. correct button press, response time) and what statistics were used to establish that the subjects were performing the task as expected (e.g. mean, range, and/or standard deviation across subjects).*

Acquisition

Imaging type(s)	*Specify: functional, structural, diffusion, perfusion.*
Field strength	*Specify in Tesla*
Sequence & imaging parameters	*Specify the pulse sequence type (gradient echo, spin echo, etc.), imaging type (EPI, spiral, etc.), field of view, matrix size, slice thickness, orientation and TE/TR/flip angle.*
Area of acquisition	*State whether a whole brain scan was used OR define the area of acquisition, describing how the region was determined.*

Diffusion MRI ☐ Used ☐ Not used

Preprocessing

Preprocessing software	*Provide detail on software version and revision number and on specific parameters (model/functions, brain extraction, segmentation, smoothing kernel size, etc.).*

Normalization | *If data were normalized/standardized, describe the approach(es): specify linear or non-linear and define image types used for transformation OR indicate that data were not normalized and explain rationale for lack of normalization.*

Normalization template | *Describe the template used for normalization/transformation, specifying subject space or group standardized space (e.g. original Talairach, MNI305, ICBM152) OR indicate that the data were not normalized.*

Noise and artifact removal | *Describe your procedure(s) for artifact and structured noise removal, specifying motion parameters, tissue signals and physiological signals (heart rate, respiration).*

Volume censoring | *Define your software and/or method and criteria for volume censoring, and state the extent of such censoring.*

Statistical modeling & inference

Model type and settings | *Specify type (mass univariate, multivariate, RSA, predictive, etc.) and describe essential details of the model at the first and second levels (e.g. fixed, random or mixed effects; drift or auto-correlation).*

Effect(s) tested | *Define precise effect in terms of the task or stimulus conditions instead of psychological concepts and indicate whether ANOVA or factorial designs were used.*

Specify type of analysis: ☐ Whole brain ☐ ROI-based ☐ Both

Statistic type for inference | *Specify voxel-wise or cluster-wise and report all relevant parameters for cluster-wise methods.*

(See Eklund et al. 2016)

Correction | *Describe the type of correction and how it is obtained for multiple comparisons (e.g. FWE, FDR, permutation or Monte Carlo).*

Models & analysis

n/a	Involved in the study
☐	☐ Functional and/or effective connectivity
☐	☐ Graph analysis
☐	☐ Multivariate modeling or predictive analysis

Functional and/or effective connectivity | *Report the measures of dependence used and the model details (e.g. Pearson correlation, partial correlation, mutual information).*

Graph analysis | *Report the dependent variable and connectivity measure, specifying weighted graph or binarized graph, subject- or group-level, and the global and/or node summaries used (e.g. clustering coefficient, efficiency, etc.).*

Multivariate modeling and predictive analysis | *Specify independent variables, features extraction and dimension reduction, model, training and evaluation metrics.*
